# Room Temperature Sensing of Volatile Organic Compounds Using Hybrid Layered SnO Mesoflowers and Laser-Induced Graphitic Carbon Devices

**DOI:** 10.1021/acssuschemeng.4c04488

**Published:** 2024-09-27

**Authors:** Richard Murray, Arbresha Muriqi, Cathal Larrigy, Alida Russo, Mintesinot Tamiru Mengistu, Daniela Iacopino, Colin Fitzpatrick, Michael Nolan, Aidan J. Quinn

**Affiliations:** † University College Cork, Tyndall National Institute, Cork, Dyke Parade T12 R5CP, Ireland; ‡ Dept of Electronic & Computer Engineering, University of Limerick, Limerick V94 T9PX, Ireland

**Keywords:** room temperature volatile organic compound sensing, laser-induced graphene, additive manufacture, resource efficient design, worker safety, density functional theory

## Abstract

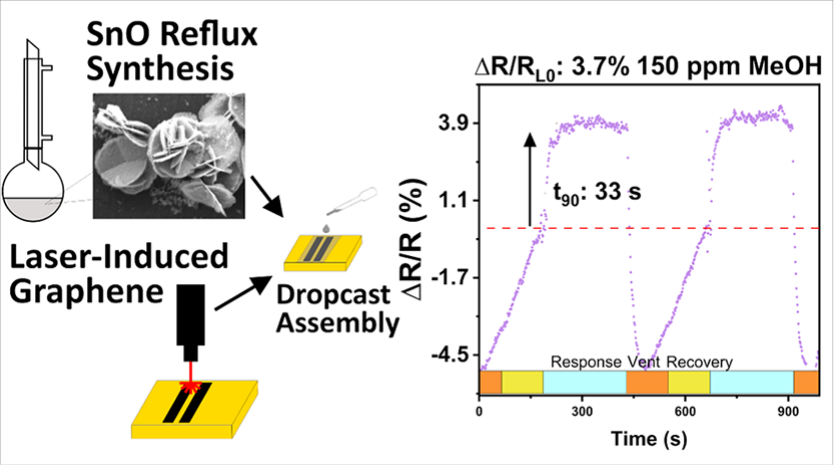

In this work, we demonstrate chemiresistive volatile organic compound (VOC) sensors prepared by drop-cast assembly of layered tin monoxide mesoflowers (SnO-MFs) on additively produced laser-induced graphene-like carbon (LIG). The SnO-MFs were synthesized below 100 °C at ambient pressure and offer a low fabrication energy alternative route to typical furnace-prepared metal-oxide materials. The additive dropcast assembly of room-temperature operating metal oxide active material allows the substitution of LIG for metal current collectors and glass for alumina, reducing the environmental footprint of the sensor. The sensors can detect methanol (150–4000 ppm) at room temperature and humidity (∼18 °C, ∼55% RH), with response and recovery times (150 ppm methanol) of *t*_90,resp_ ≈ 50 ± 10 s and *t*_90,rec_ ≈ 5 ± 0.5 s, respectively. The sensors demonstrated a limit of detection (170 ± 40 ppm) below 8 h worker safety exposure levels (200 ppm) and stable DC resistance responses Δ*R*/*R* = 9 ± 2% to 710 ppm of methanol for over 21 days in ambient laboratory conditions, *n* = 4. First-principles density functional theory simulations were used to elucidate the interactions of VOC species on the SnO surfaces. LIG-SnO hybrid sensors thus present a resource-efficient route to develop chemiresistive sensors for low-power applications, although with cross-selectivity to other alcohol species.

## Abstract 2

Reflux preparation of layered SnO flowers,laser-induced graphene preparation, dropcast assembly of VOC sensors,and associated response to MeOH exposure.

## Introduction

Many volatile organic compounds (VOCs) are released during industrial processes and in domestic settings; as byproducts of combustion, fuel storage, cooking, or through outgassing from household furnishings.^1−6^ They can build up in enclosed spaces resulting in increased local concentration and associated cumulative exposure risk.^7,8^ VOCs can pose a significant health risk at concentrations below human perception, e.g., methanol 8 h worker safety threshold (200 ppm).^9−12^ Thus, reliable VOC sensors are necessary for worker and domestic safety through integration with the Internet of Things, either as worker wearables or as part of sensor networks within a workplace or domestic setting.

However, conventional VOC sensors typically use semiconductor-manufactured electrode platforms formed from critical raw materials. The functional MOX materials typically require high-temperature processing steps to form the correct material phase and/or to calcinate previously synthesized MOX nanoparticles (Table 1), again, increasing the environmental footprint impacts.^14,15^ Further, conventional MOX VOC sensors often require elevated operating temperatures (>300 °C), which poses significant challenges for low-power sensors for wearable or wireless sensor applications and further increases the environmental footprint. The net result of which is that current chemiresistive VOC sensors have high environmental footprint impacts (global warming potential, resource depletion, and human- and eco-toxicity).^13^

**Table 1:** Integration Challenges for Existing Chemiresistive MOX VOC Sensing Morphologies

Assembly Mode	Electrode	Support	Sensing Material Formation Temp. (>300 °C)	Post calcination (>300 °C)	Contains Critical Raw Materials^a^	Elevated Operational Temp. (>300 °C)	ref
Thin Film	Prepatterned Metal	Ceramic	Yes	No	Yes	Yes	(19−23)
Thick Film	Prepatterned Metal	Quartz	Yes	Yes	Yes	Yes	(24−28)
Sintered Nanoparticles	Prepatterned Metal	Ceramic	Yes	Yes	Yes	Yes	(14,29−32)
Self-assembled particles	LIG Electrode	Polymer	No	No	No	No	This work

a Critical raw materials identified from the Study on the EU’s list of Critical Raw Materials (2023), e.g., Pt, Pd, Rh, Ti, and W.^13^

Extensive work to enhance chemiresistive sensor performance (response magnitude, sensitivity, selectivity, stability, and operational temperature) has been carried out in the literature to date, including the decoration of semiconductor MOX with precious metal catalysts;^11−14^ mixed-deposition of MOX nanoparticles;^10,15−19^ miniaturization;^20−22^ high-aspect-ratio structures;^23−25^ use of hollow spheres;^26−28^ and molecularly imprinted polymers,^29^ among other approaches. This is outlined more completely in Section SA. However, the improvements yielded by these methods typically come at the cost of sustainability of the resulting sensor.

Quantitative “cradle-to-grave” life cycle analysis (LCA) and assessment of environmental footprint impacts is challenging for emerging research materials and fabrication processes at low technology readiness levels due to a lack of available and/or standardized data.^16,17^ Many early stage sustainability assessments use comparative or streamlined approaches for a subset of life cycle stages and also consider a limited number of environmental footprint impact categories to focus on “hotspots,” which dominate the overall environmental footprint. In particular, global warming potential or, as a proxy, cumulative energy demand, is considered particularly useful, given the strong correlation with other environmental impacts.^18^ With this in mind, this paper reports on a new approach to reduce the environmental footprint of chemiresistive MOX VOC sensors by addressing three key hotspots identified for conventional MOX devices: Fabrication of the MOX sensing material; electrode fabrication and MOX material integration; and the MOX sensor operating temperature.

**Figure 1:**
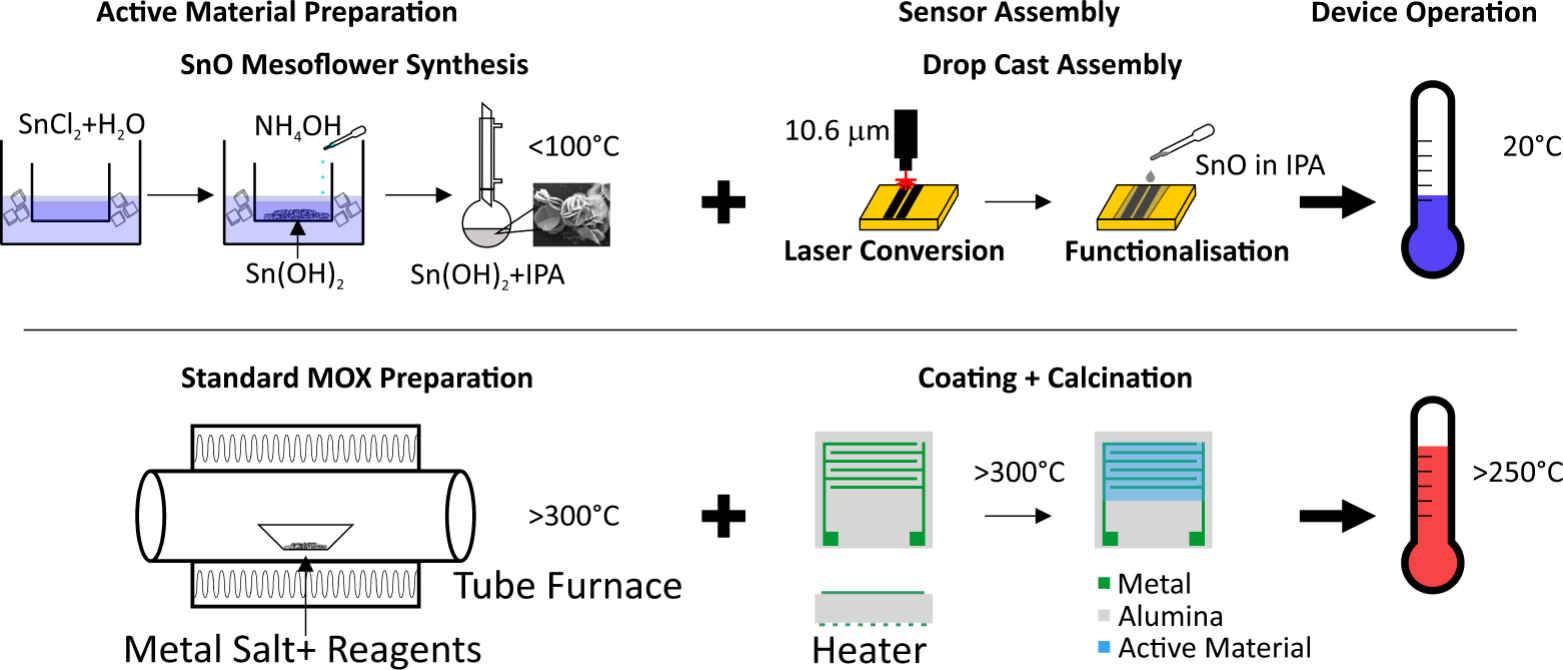
Schematic of VOC sensor preparation route. (Top) Reflux particle synthesis and dropcast assembly on LIG. (Bottom) tube furnace synthesis of MOX particles and calcination assembly on lithographically defined electrodes.

Table 1 compares our approach to reported literature results for MOX VOC sensors, and Figure 1 illustrates our proposed improvements over the fabrication and operation life cycle stages. Our approach addresses all three hotspots.

For MOX material fabrication, we utilize a low-temperature (<100 °C) reflux synthesis route for the preparation of polycrystalline-layered SnO mesoflowers (SnO-MF) vs conventional devices based on tin dioxide (SnO_2_) nanoparticles or films. SnO is a layered material, due to the Sn 5*s* lone pair distorting its crystal structure, allowing for enhanced surface/volume ratios and accessible 5*s* orbitals.^33−35^ This, in addition to its *p*-type semiconducting nature due to Sn vacancies, and its metastability up to moderate temperatures (370 °C), make it an attractive candidate for room-temperature VOC sensing.^34,36−38^ To date, there have been limited investigations using SnO, with most reports focusing on SnO_2_ due to its well-established synthesis routes and its stability. However, the requirement for high-temperature (400–800 °C) preparation and calcination process steps for SnO_2_ increases the cumulative energy demand and therefore global warming potential.^39^ Simple, scalable reflux chemistry routes using green solvents to produce SnO particles suitable for VOC detection with reduced energy cost have been reported with reflux-solvent-controlled particle morphology.^40^

For the contact electrodes, laser-induced graphitic carbon (LIG) is an attractive candidate electrode material for replacing traditionally manufactured metal electrodes. Discovered in 2014, this laser graphitization method enables site-specific direct-write formation of highly porous, three-dimensional, conductive carbon electrodes on suitable polymer substrates through formation of a laser plasma, even under atmospheric conditions.^41^ LIG was initially formed on polyimide, a synthetic polymer, by using a 10.6 μm CO_2_ laser. LIG formation has also been reported using a range of laser wavelengths,^42^ other synthetic and biopolymer feedstock substrates,^43−46^ and with resource-efficient strategies for performance optimization and reliable electrical contacting.^47,48^ Proof of concept demonstrations have been reported for energy storage,^45^ electrochemical sensing,^49,50^ and more recently for gas sensing.^51−54^

**Figure 2:**
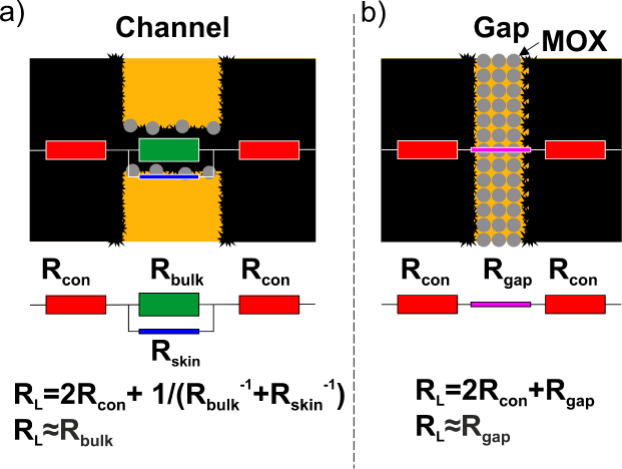
Schematic (not to scale) showing response sensitivity dependence on device functionalization geometry for (a) channel and (b) gap devices.

To date, most LIG-based gas sensors have focused on detection of reactive oxidizing gases (NO, NO_2_), both as resource-efficient contact electrodes,^51,52^ and as active material.^53,54^ The majority of these works have investigated resistive LIG channel devices (Figure 2a), both as-fabricated,^54^ and with MoS_2_ functionalization.^53^ While these channel devices showed strong responses in dry synthetic air, they showed significant humidity effects and long response/recovery times (>113–580 s). The channel geometry also means that the total device resistance (*R*_T_) is dominated by the resistance of the LIG channel, leading to small resistance changes when exposed to target VOCs, compared to equivalent works on decorated gap geometries (<1% vs >10%). Moreover, Mo is a critical raw material, which may pose challenges for widespread deployment. These factors informed our device architecture choice in using SnO as the active material with LIG contact electrodes (Figure 2b), where the overall device resistance is dominated by the resistance of VOC-sensitive SnO-MF in the gap.

Finally, our devices operate at room temperature vs the elevated temperatures needed for conventional chemiresistive MOX VOC sensors (>300 °C), thereby reducing energy consumption and obviating the need for resource- and energy-intensive substrates. Our approach shows that development of future chemiresistive VOC sensors will require “4-S co-optimization”, to provide acceptable levels of sensitivity, selectivity, stability, while ensuring sustainability, particularly for widely deployed or short-lifetime devices.

## Experimental Section

### Device Fabrication

#### Laser Graphitization

Adhesive-backed polyimide tape (Radionics HB830, 70 μm thickness) was drawn and affixed to clean glass slides, ensuring that no air bubbles were trapped. The polyimide top surface was converted to laser-induced graphene by CO_2_ laser raster patterning at 4.2 W average laser power, ∼200 mm s^–1^ scan speed (1000 pixels per inch, PPI) and ∼20 lines mm^–1^ raster line density (Universal Laser system, PLS4.75, 10.6 μm, 30 W max. average laser power). Smooth edges were defined by a vector write (3.7 W average laser power, ∼ 200 mm s^–1^ scan speed, 1000 PPI), yielding two parallel rectangular carbon electrodes (10 mm × 3 mm) separated by an ∼100 μm interelectrode gap.

#### Particle Synthesis

SnO-MF were synthesized following the method of Jaśkaniec et al.^40^ Anhydrous SnCl_2_ (7.56 g, Merck, 98%), was dissolved in deionized (DI) water (18.2 MΩ cm, 400 mL) in an ice bath. Ammonium hydroxide (NH_4_OH, 10 mL, 28%, Merck) was added dropwise while being stirred, yielding a white precipitate, which was centrifuged, and then washed with DI water and isopropyl alcohol (IPA, Merck, 99.7%). 3 g of this precipitate was dispersed in 60 mL of IPA and refluxed while stirring in a mineral oil bath at 90 °C for 18 h until a brown/black dispersion was produced, which contained the SnO-MFs.

#### Device Fabrication

A 250 μL aliquot of the as-produced SnO-MF dispersion was added to 2 mL of IPA. 25 μL aliquot of the dilution was then drop-cast onto the as-fabricated LIG electrodes and allowed to dry overnight, yielding the VOC sensors. These devices were electrically contacted with mechanical clips.

### Material Characterization

#### Raman

Raman spectra were acquired at multiple regions across the functionalized laser-induced graphitic carbon–SnO hybrid sensor (Horiba XploRA Raman microscope, 532 nm, 70 mW, 50× objective, 2400 lines/mm sample grating, 60 s sampling time, 1 accumulation, 1% filter). Spectra are reported without background subtraction or smoothing.

#### XRD

Precipitated SnO-MFs were dried and deposited onto double-sided tape on microscope slides to produce a powder film. θ–2θ powder X-ray diffraction scans were acquired between 20° and 80° (PANalytical X’pert PRO XRD, copper anode, Kα_1_ = 1.5406 Å, Kα_2_ = 1.5444 Å, 1/2° divergence slit, generator voltage 45 kV, 40 mA).

#### Microscopy

Scanning electron microscopy (SEM) images of LIG SnO-MFs, covered with ∼6 nm of Au to reduce charging effects, were acquired by using an FEI Quanta 650 scanning electron microscope (10 kV beam voltage).

### Gas Sensing

We developed a simple system for headspace VOC vapor detection from multiple SnO-LIG sensors under controlled humidity conditions; see Section SC.3. This resource-efficient system would be within the budget of many undergraduate teaching laboratories.

#### Experimental Setup for Headspace Vapor Detection

Time-controlled venting using humidified nitrogen with consistent pressure and relative humidity was achieved by using an Arduino-controlled solenoid valve interfaced to a dry nitrogen line divided into two paths, each connected to a flow controller. One line was connected to a bubbler containing DI water, with check valves to prevent backflow, and then to a condenser. The humidified condenser output line and the dry nitrogen line were rejoined in a mixer. The relative humidity of the nitrogen line was controlled by changing the ratio of the gas flows at the flow controllers to match ambient room humidity (∼55–65% RH) as verified by a humidity detector (RS-192TK Temperature and Humidity Datalogger). Further detail is provided in Section SC, Figure S4.

#### Gas Sensing

The functionalized sensors were mounted in a 3D-printed sample holder (Figures S2 and S3) and electrically contacted with a maximum of 6 sensors inside a glass chamber (∼600 mL volume). A 100 MΩ balancing resistor was connected in parallel to each device, Figure S5, to reduce the equivalent circuit resistance below the limit of the multichannel multimeter used (Keithley DAQ 6510, 7700-20 slot card). Devices were measured simultaneously at laboratory room temperature (∼18 °C) via multiplexing with 5 averaged readings per measurement (number of power line cycles, NPLC = 5), time interval between measurements: 100 ms, and the multimeter measurement signal was verified as 0.4 V DC with a 0.25 V amplitude AC modulation (50 Hz) with an oscilloscope (Tektronix TDS 220).

Cycles of humidified nitrogen gas vents and liquid phase solvent additions (1 μL–50 μL) were carried out using microsyringes (Hamilton, 10 μL/50 μL/100 μL) while monitoring the device resistance to determine the solvent response. Liquid phase aliquots of target individual VOCs (methanol (MeOH) (99.8%, Merck), ethanol (EtOH) (99.9%, Ocon), isopropanol (IPA) (99.5% Merck), acetone (ACE) (99.5%, Merck) were injected into the chamber and the corresponding VOC vapor concentration (in ppm) was measured at specific time intervals using a photoionization detector (Tiger PID, 11.7 eV lamp), standardized against a calibrated reference gas (100 ppm isobutylene in balance air), see Section SC.5.

#### Aging Study

Sensors were mounted in the sample holder and electrically contacted. Their sensing responses to 1 μL of MeOH (∼150 ppm) and 5 μL of MeOH (∼710 ppm) were recorded on the initial and subsequent days. The laboratory temperature and humidity were recorded each day, and the vent humidity was set to match the laboratory humidity (∼55–65% RH).

### Computational Methodology

Density functional theory (DFT) calculations were employed to investigate the interaction of MeOH, EtOH, IPA, ACE and water at two specific low-energy facets of SnO: (001) and (101).^40,55^ Calculations were conducted using a periodic plane-wave basis set implemented in the Vienna *Ab initio* Simulation Package (VASP) version 5.4.^56^ Core electrons were treated through the projector augmented wave (PAW) method, and the valence electronic configurations used were: Sn: 5*s*^2^5*p*^2^, O: 2*s*^2^ 2*p*^4^, C: 2*s*^2^2*p*^2^, and H: 1*s*^1^.^57^ We employed the Perdew–Burke–Ernzerhof (PBE) approximation to the exchange–correlation functional.^58^ The plane wave cutoff was 400 eV, and a Monkhorst–Pack sampling *k*-point grid of (3 × 3 × 1) was used for both surfaces. The force convergence criteria was set at 2 × 10^–2^ eV Å^–1^, while the energy convergence criteria was 1 × 10^–4^ eV. The equilibrium lattice parameters for all SnO models from this setup are *a* = *b* = 7.59 Å, *c* = 24.03 Å, and α = β = γ = 90° for the SnO (001) surface and *a* = 12.39 Å, *b* = 7.62 Å, *c* = 23.93 Å, and α = β = γ = 90° for the SnO (101) surface. The atom counts for the surface models are 44 for SnO (001) and 48 for SnO (101). For the models with molecules adsorbed on top, the atom count of the organic/water molecule is added to these numbers.

Interaction energies of the target molecules were calculated by using the following equation:
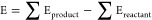
1

Where ∑E_product_ is the summed energy of the product species and ∑E_reactant_ is the summed energy of the reactant species.

## Results and Discussion

### Material Characterization

We have focused on methods to reduce the environmental footprint of chemiresistive VOC sensors by combining resource-efficient LIG current collectors with the low mass loading of resource-efficient semiconducting SnO mesoflowers.

**Figure 3:**
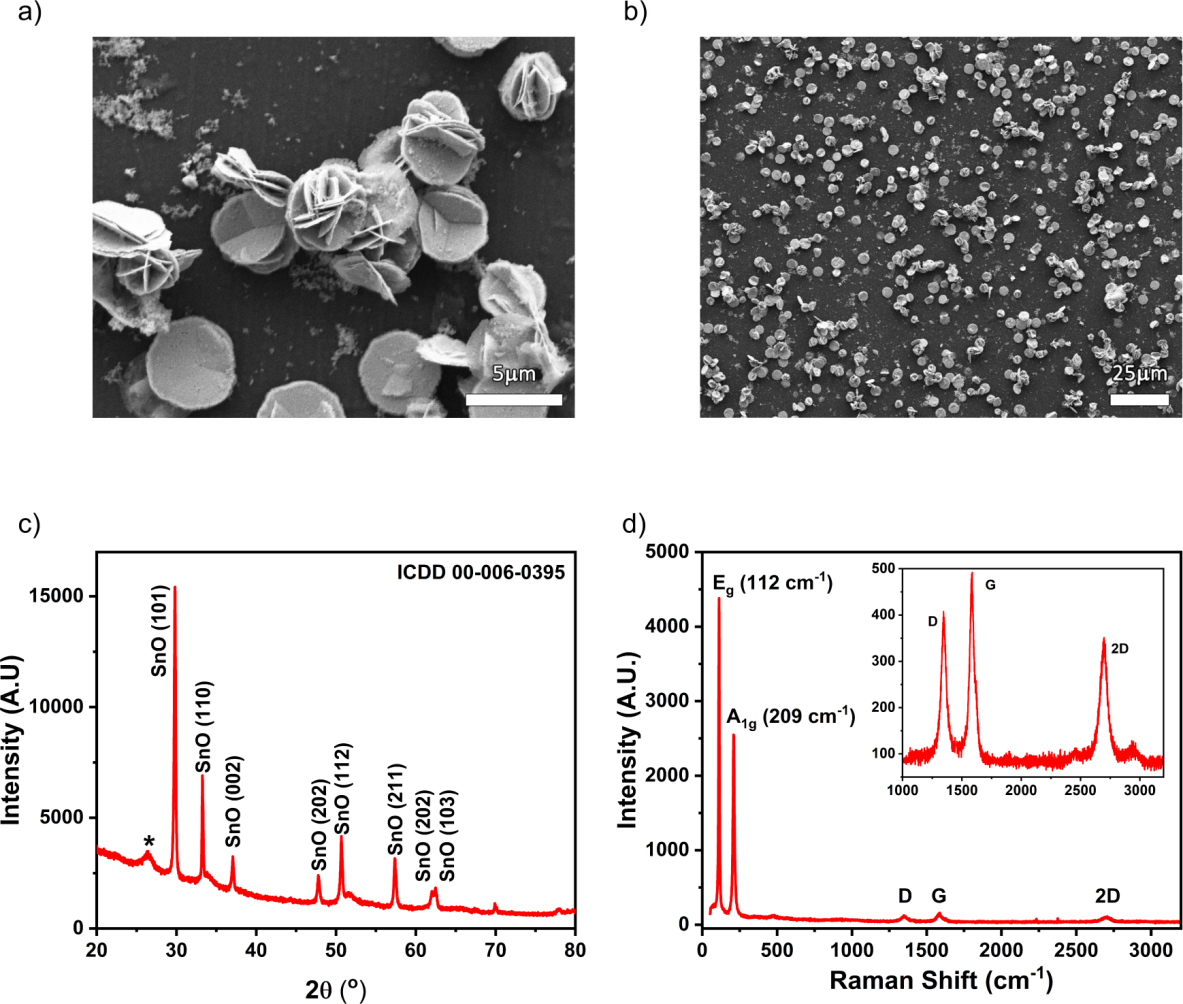
(a) Scanning electron micrograph demonstrating the SnO-MF flower-like morphology. (b) Scanning electron micrograph of SnO-MFs dropcast on polyimide film displaying disordered, random aggregation. (c) Powder X-ray diffractogram of as-synthesized SnO-MFs on a supporting glass slide. (d) Raman spectrum of SnO NP on the LIG electrode surface with Raman spectrum of the LIG electrode (inset).

Figure 3 shows the microstructure, crystal phase, and oxidation state of the synthesized SnO-MFs. Figure 3a demonstrates the expected interlocked disc structure for particles produced in IPA.^40^ The quasiflower morphology comprises intersecting micrometer-scale disks with diameters of 4.0 ± 0.5 μm and thickness ≈ 400 nm (Figure S7). The SnO particles are formed from the reflux of intermediate Sn_6_O_4_(OH)_4_, initially prepared from SnCl_2_ under basic conditions. In the reflux in IPA, and at these modest synthesis temperatures, <100 °C, SnO forms through a complex dissolution–reprecipitation process, whereby the amorphous intermediate Sn_6_O_4_(OH)_4_ is consumed to produce nanoscale platelets that aggregate into larger structures, in this case, a quasiflower made of platelets.

More generally, the resulting aggregate structure is determined by several factors; including, the ability of the solvent to stabilize different crystallite planes, its surface tension, the influence of the solvent pH of dissolution rate, the temperature of the reflux and its disorganizing influence on aggregation kinetics, purity of the solvent, and presence of oxygen or capping agents to name a few contributions. These can be used to tune the structure of particles produced and act as an exciting route to further optimize the sensing performance.^39,40,51,59−63^

XRD data of a dense film of SnO-MFs (Figure 3c) shows peaks characteristic of the expected romarchite phase of SnO (ICDD 00-006-0395, space group *P*4/*nmm*), with an additional broad peak at ≈26.4° marked with an asterisk associated with the glass substrate used. This peak is not present for scans acquired for SnO mesoflowers on a Si (001) wafer, as shown in Figure S10. Raman data (Figure 3d) of SnO-MFs deposited on LIG show the expected peaks for SnO: *E*_g_ (112 cm^–1^) and *A*_1g_ (209 cm^–1^), together with weaker *D* (1347 cm^–1^), *G* (1585 cm^–1^) and) 2*D* (2695 cm^–1^) peaks typical of good quality LIG.^47^ No peaks corresponding to SnO_2_ formation were observed, similar to the XRD data.^64^

Raman data reported by Saji et al. for few- to many-layer SnO films prepared by pulsed laser deposition revealed a quasi-linear decrease in the ratio of the areas under *A*_1g_ and *E*_g_ peaks as the number of SnO layers increased.^65^ We found *A*_1g_/*E*_g_ peak area ratios ranging from 1.35 to 1.94, in reasonable agreement with the reported range for bulk SnO (1.65) down to 12-layer films (1.95).

From Lorentzian fits to the (101) XRD peak (Figure 3c), the Scherrer formula yields a mean crystallite size of ≈1.1 nm in the direction perpendicular to the plane. This suggests that the observed petal thickness (≈100 nm) arises from polycrystalline aggregation of nanoparticle building blocks, as previously reported for SnO.^61^ Thus, the nanostructured surface enhances the surface area–volume ratio, increasing the density of potential adsorption sites, while the *p*-type semiconductor nature of SnO, verified by a Mott–Schottky plot (Figure S8), should result in decreased conductivity upon electron transfer.^66−68^ Given that many VOCs are reducing gases (electron-donating), the resistance through the particle network should, therefore, increase upon VOC exposure.

### MOX Quasiflower Directed Assembly and Mode of Operation

Figure 3b demonstrates that deposition of low mass loadings of SnO-MFs on polyimide produces sparse clusters, i.e., no effective conduction pathways. Fortuitously, LIG produced by raster-scanning a CO_2_ laser over the polyimide substrate results in some lateral growth at electrode edges and redeposition of graphitic material in the interelectrode gap.^47^ While these features may limit the lateral critical dimensions of LIG devices, they also offer a route to promote the local aggregation of SnO-MFs and enable the formation of hybrid LIG-SnO-MF conducting networks with minimal SnO-MF mass loading, thereby improving resource efficiency.

**Figure 4:**
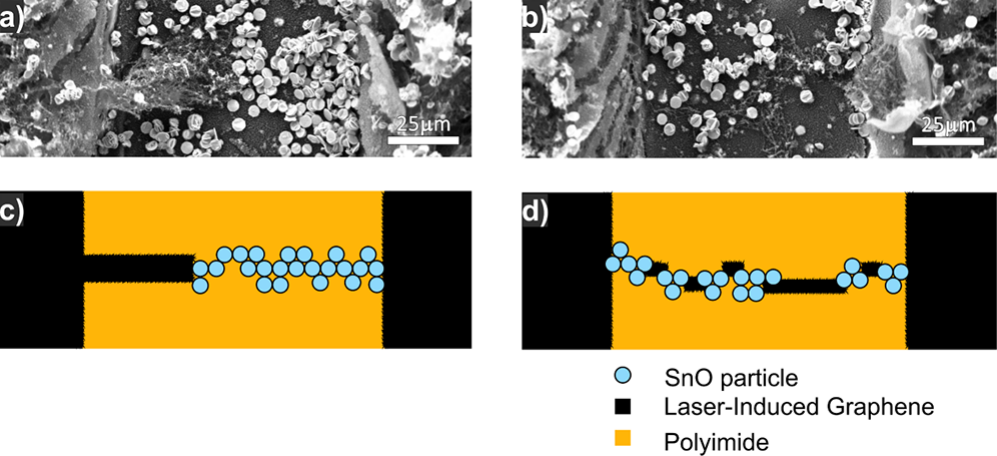
(a) Scanning electron micrograph (SEM) of a potential conduction pathway featuring a LIG bridge in series with aggregated SnO-MFs. (b) SEM of a pathway comprising LIG “stepping stones” linking SnO-MF aggregates. (cd) Schematics corresponding to (ab), respectively.

Figure 4 shows examples of hybrid-SnO-LIG electrode bridging conformations in the interelectrode gap. The carbon inclusions can act as conductive elements that reduce the effective gap width (Figure 4a) or scaffolds that promote aggregation of SnO-MFs following drop deposition and solvent evaporation (Figure 4b). Devices then consist of multiple, separate LIG-SnO conduction channels (1 + 1 D network). The dominant conduction pathways can be viewed as either a LIG “bridge” (Figure 4c), which connects the contact electrode to a SnO-MF aggregate, or LIG “stepping-stones” (Figure 4d), which connect multiple separated SnO-MF aggregates. Other pathways and failure modes are outlined in Figure S10. Since the LIG structures show much lower resistivity (∼10^–3^ Ω m) than the SnO-MFs (intrinsic *p*-type semiconductors), conduction through these hybrid paths will be dominated by charge transport through SnO-MFs.^47^ IV characterization from −10 to 10 V, shown in Figure S9., indicates a slight deviation from ohmic behavior, in line with the dropcast self-assembly of a granular network and Schottky barrier formation at the LIG current collectors.

### Sensor Performance

**Figure 5:**
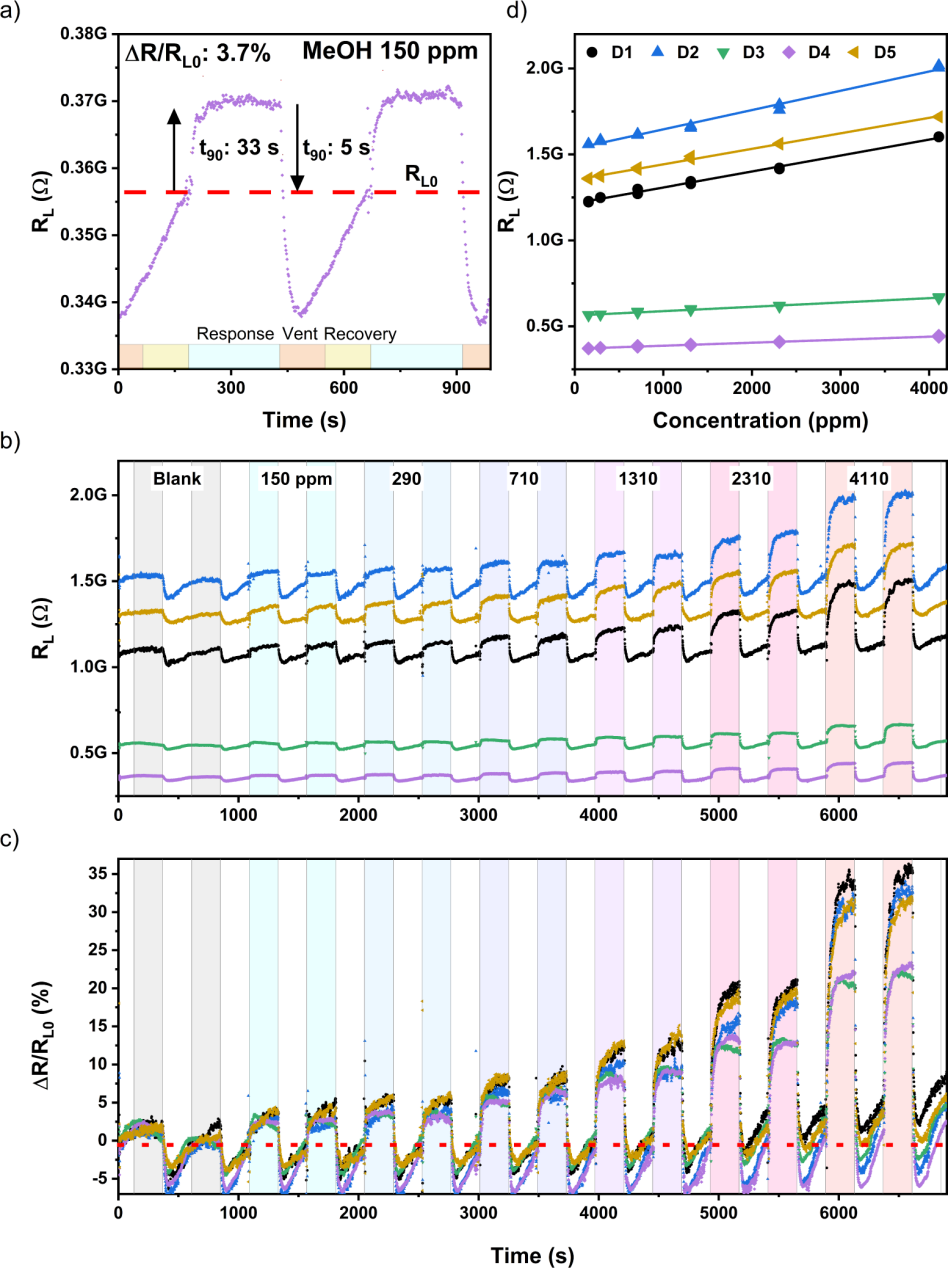
(a) Sensing response behavior to MeOH exposure, demonstrating *p*-type response and *t*_90_ response and recovery times of 50 ± 10 and 5 ± 0.5 s, respectively. (b) Resistance–response curves for five sensors monitored simultaneously during a sequence of increasing MeOH concentration (150–4110 ppm), colored bars highlight the addition of MeOH. (c) Percentage–response curve of data shown in panel (b), demonstrating similar response behavior. (d) Calibration curve of simultaneously monitored sensors responding to MeOH solvent additions.

A custom gas sensing chamber was designed to mimic the headspace detection of VOC vapors at ambient temperature (∼18 °C). Figure 5a depicts the changes in the DC resistance (*R*_L_) of one of the hybrid SnO-LIG VOC sensors in the chamber during two separate injections of MeOH aliquots. Prior to addition, the sensing chamber (≈0.6 L volume) is purged with humidified nitrogen for 2 min (>20 L/min., ∼55–65% RH, matched to lab. humidity)—“Vent” period in Figure 5a. The system is allowed to equilibrate for 2 min (“Recovery”). An aliquot of the VOC solvent (1 μL MeOH here) is added using a microsyringe and evaporates after landing on the heated chamber base. The response value, Δ*R*_L_, is taken as the resistance increase ≈3 min after VOC addition, corresponding with the steady-state measurement of gas phase VOC concentration using a photoionization detector, see Figure SC5. Figure 5a also illustrates the rapid response times for these room-temperature sensors, with measured *t*_90,resp_ response time constants, defined as the time taken to rise to 90% of the maximum resistance response, of *t*_90,resp_ ≈ 50 ± 10 s for 1 μL (150 ppm) of MeOH. The corresponding recovery time constant, i.e., time taken for resistance to fall by 90% during purging, was even shorter, *t*_90,rec_ ≈ 5 ± 0.5 s.

Figure 5b demonstrates the reproducibility of 5 sensors simultaneously exposed to a sequence of MeOH aliquot injections, from 150 ppm (1 μL aliquot) to 4110 ppm (50 μL aliquot). The sixth device in the chamber showed unstable behavior (Figure SC5.4). Each run begins with one or more “blank” cycles to measure baseline behavior without introducing the VOC solvent. Figure 5c shows the normalized percentage response (Δ*R*_L_/*R*_L0_) for the devices with data shown in Figure 5b, where *R*_L0_ is taken as the device resistance measured before the first injection of MeOH. Significant response values (Δ*R*_L_/*R*_L0_ ≈3–5% are observed for low concentrations (150 ppm), increasing to Δ*R*_L_/*R*_L0_ > 20% for higher concentrations (4110 ppm). Similar percentage response magnitudes and time dependences were observed for the 5 devices shown in Figure 5b, despite differences in baseline resistance. The slight increase (<1.5%) in baseline resistance observed at higher MeOH concentrations (≥4110 ppm, Figure 5b) was possibly due to incomplete purging. However, since the saturation concentration of the MeOH headspace was measured under identical conditions (Figure S6), the measured response accounts for this cumulative buildup at larger aliquot volumes.

The MeOH response versus concentration data for these sensors is presented in Figure 5d, with corresponding limits of detection (LOD) extracted from linear fits tabulated in Table 2. The mean LOD for MeOH measured across the 5 devices, 170 ± 40 ppm, is below the 8-hour OSHA and EPA worker safety limit of 200 ppm.^9−11^

**Table 2:** Limit of Detection of MeOH and EtOH Extracted from Figures 5d and 7c Respectively

Solvent	Device 1 LOD (ppm)	Device 2 LOD (ppm)	Device 3 LOD (ppm)	Device 4 LOD (ppm)	Device 5 LOD (ppm)	Mean LOD (ppm)
Methanol	149 ± 3	225 ± 8	167 ± 4	197 ± 6	122 ± 2	170 ± 40
Ethanol	210 ± 9	188 ± 8	106 ± 2	205 ± 9	179 ± 7	180 ± 40

The device-to-device variability in baseline resistance data (Figure 5c) reflects the proposed conduction mechanism through self-aggregated SnO-MFs partially bridged by LIG. As noted above, the sensors show consistent response magnitude, shape, and time dependence. For some devices with a high degree of carbon bridging (lower initial resistance), the sensor becomes less sensitive at high MeOH concentrations (Figure S16) suggesting that the degree of carbon bridging could provide a viable route for future optimization.

The MeOH addition response time is shown in Figure 5a. The time taken to rise to 90% of the maximum response (*t*_90,resp_) was *t*_90,resp_ ≈ 50 ± 10 s for 1 μL (150 ppm) of MeOH. The *t*_90,resp_ for a 50 μL (4110 ppm) addition of MeOH was 67 ± 7 s and is presented in Figures S11 and S12. It is likely that the simple liquid phase addition–evaporation method employed here to generate headspace VOCs aliases the measured response time with the evaporation rate. Therefore, we consider the measured response times to be the upper bound. The recovery time, ≈5 ± 0.5 s, was unaffected by increased VOC concentration levels. We note that the solvent introduction method employed here enables a resource-efficient study of a variety of VOC compounds and serves as a versatile pathfinding route to assess new sensor configurations.

**Figure 6:**
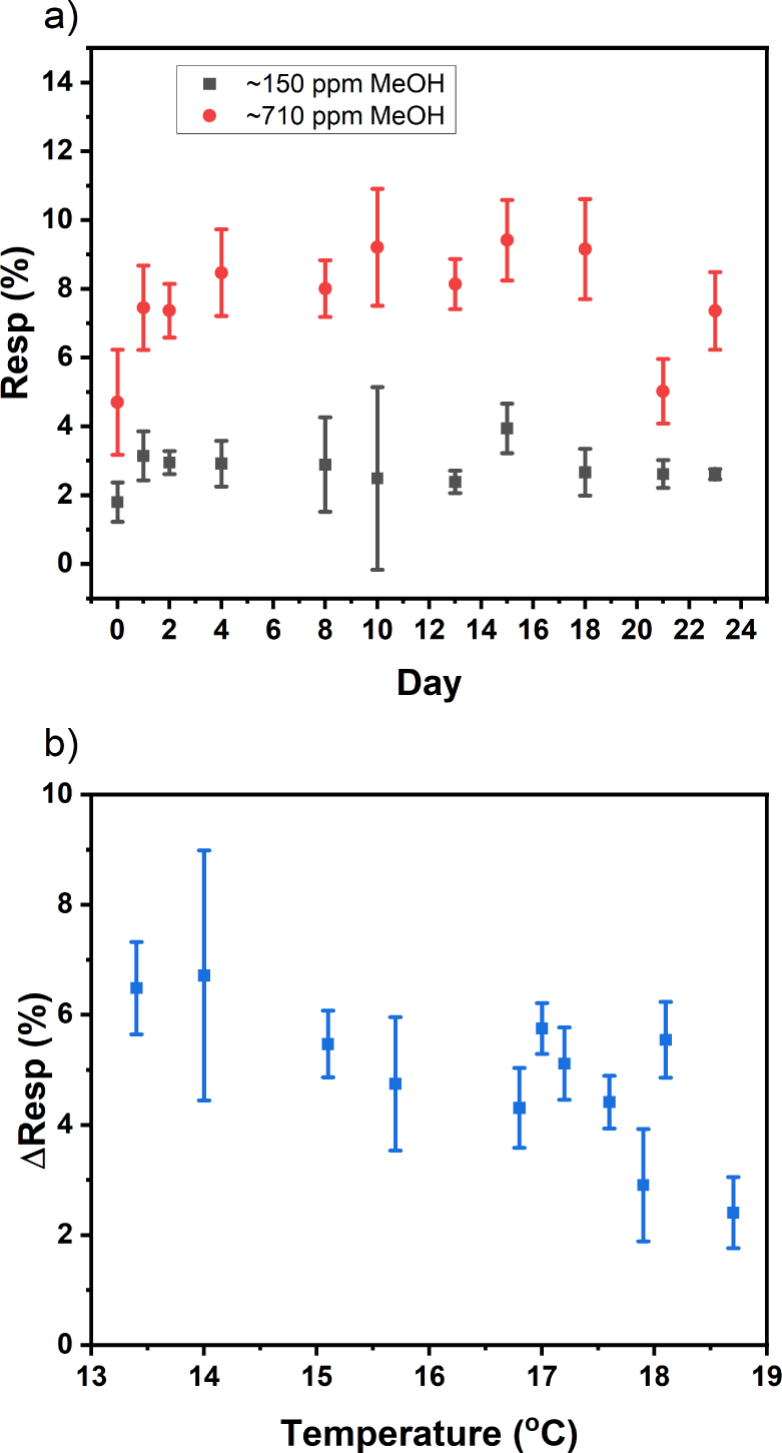
(a) Mean (*n* = 4) response data over a 23-day period toward 1 μL MeOH (150 ppm), *Resp*_150 ppm_; and 5 μL MeOH (710 ppm), *Resp*_710 ppm_. (b) Mean (*n* = 4) dependence of response difference, Δ_Resp_ = *Resp*_710 ppm_ – *Resp*_150 ppm_, vs ambient laboratory temperature.

Figure 6 shows the stability assessment of a set of four SnO-LIG hybrid VOC sensors to injected MeOH aliquots (1 μL, 5 μL) once per day across 22 days, excluding weekends. Each measurement cycle comprised (vent/wait, blank) × 2; (vent/wait, 1 μL MeOH) × 2; (vent/wait, 5 μL MeOH) × 2; vent. The chamber was then left to recover with the inlet valve closed and the outlet open under ambient conditions. The sensors demonstrated stable average values of the DC resistance responses across the 4 sensors to 5 μL aliquots of MeOH (∼710 ppm) over the 22 day period, *Resp*_(710 ppm)_ = Δ*R*/*R*_0_ = 9 ± 2%. The observed device variability reflects changes in the ambient laboratory temperature and humidity over the period (Figure S19). To gain more insight, we define the response difference for each individual sensor to injections of 5 μL (710 ppm MeOH) and 1 μL (150 ppm MeOH), respectively, as
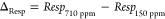
2

Figure 6b shows a quasi-linear decrease in Δ_Resp_ with increasing temperature (13–19 °C). This further supports our assertion that charge transport near room temperature is not dominated by thermally induced oxygen radicals.

**Figure 7:**
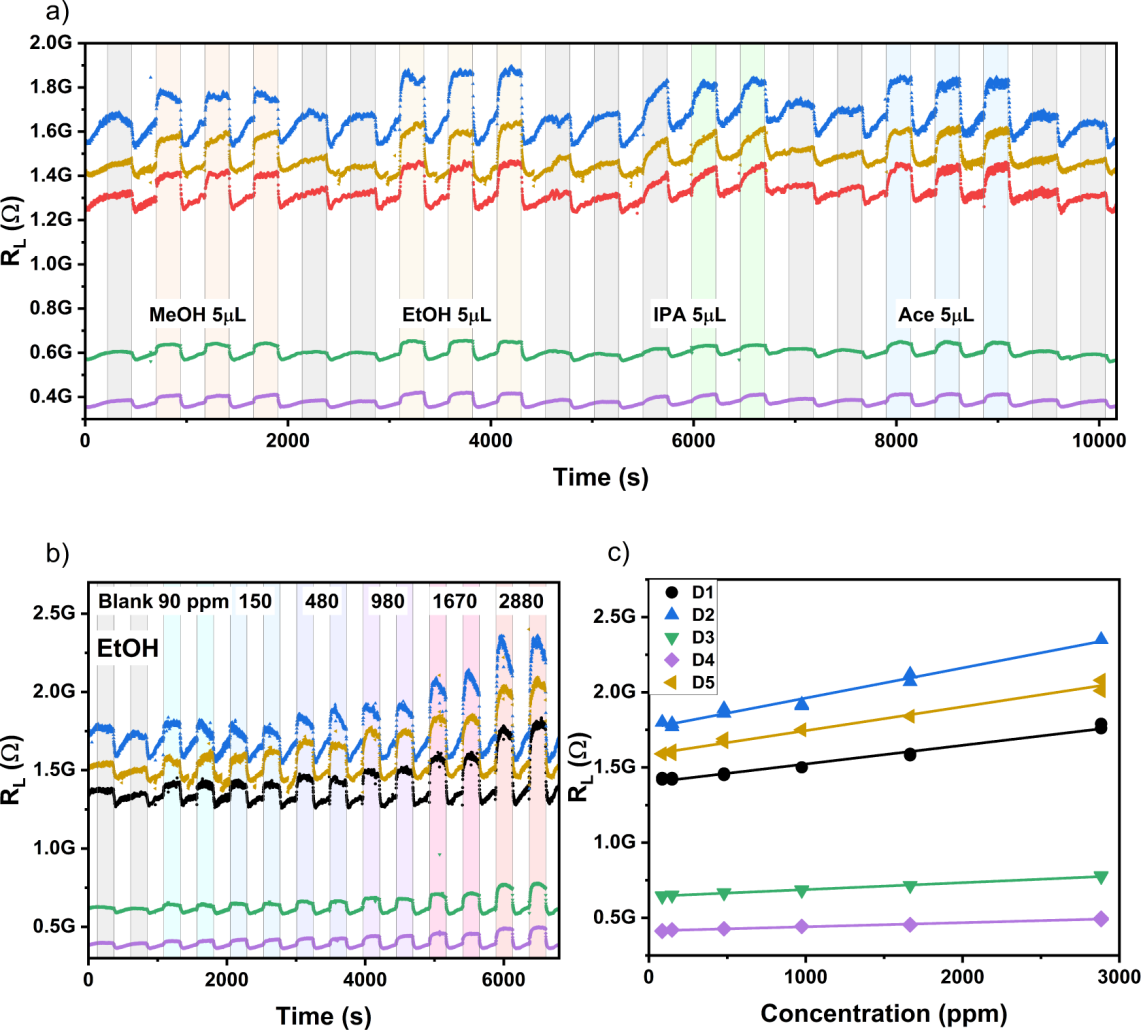
(a) Cross-selectivity response toward 5 μL of MeOH, EtOH, IPA, and ACE. (b) Response curve for five VOC sensors simultaneously exposed to the same EtOH concentration ramp, colored bars highlight the addition of EtOH. (c) Calibration curve of simultaneously monitored sensors responding to EtOH solvent additions, demonstrating a mean limit of detection < 200 ppm.

Figure 7a shows the cross-selectivity behavior to EtOH, IPA and ACE. We observe comparable response (Δ*R*_L_/*R*_L0_) values for 5 μL aliquots of MeOH (8.3 ± 1.0%), EtOH (12.5 ± 1.9%), and ACE (8.8 ± 0.7%). The response data for IPA demonstrate incomplete recovery behavior, suggesting a high adsorption affinity at the SnO-MFs. This is not unexpected, given that IPA was the reflux solvent for their synthesis. Figure 7b shows the response data vs time for an EtOH concentration ramp for our LIG-SnO-MF devices, and Figure 7c shows the corresponding calibration curves. The mean extracted LOD for EtOH is 180 ± 40 ppm (Table 2), similar to the mean LOD for MeOH, 170 ± 40 ppm.

Table 3 compares the performance of the SnO-MF LIG sensor to examples of SnO and SnO_2_ sensors and to LIG-based sensors. However, there remains limited characterization of these materials and systems toward alcohols. The majority of these systems instead focus on NO and NO_2_ due to their high toxicity at low concentrations, and associated large response magnitude per molecule, caused by their large electron-withdrawing affinity. The SnO-MF LIG sensor performs well when compared to the state-of-the-art Sn-MOX sensors, with comparably rapid response/recovery times as higher energy fabrication routes such as calcined MIP decorated SnO_2_, despite its modest reflux synthesis and room temperature operation. Similarly, as outlined in the introduction and Figure 2, the SnO-MF LIG sensor demonstrates a response expected of a modified gap sensor, whereby the system resistance is dominated by the gap region, allowing for its modulation upon VOC exposure. In this instance, due to the highly resistive nature of *p*-type SnO, it is unlikely that any measurable change in resistance would have been detected if a channel geometry had been used. This demonstrates the potential for direct use of lower-resource MOX materials for room temperature VOC sensing.

**Table 3:** Comparison between SnO-LIG Sensors, Sn-Sensors, and LIG-Based Sensors

Sn-Based Sensors							
Active Material	N/A	Analyte	Conc. (ppm)	Temp. (°C)	Response (%)	Resp/Rec Time	ref
Frame-like SnO (solvothermal)		NO_2_	1	200	275%	30/30 s	(63)
SnO/SnO_2_ (sputtered)		NO_2_	10	60	435%	2.75/5.5 min	(69)
SnO_2_ + MIPS (Hydrothermal)		MeOH	100	220	616%	12/55 s	(70)

LIG-Based Sensors and LIG-Electrode Sensors							
Active Material	Geometry	Analyte	Conc. (ppm)	Temp. (°C)	Response (%)	Resp/Rec Time	ref
MoS_2_ Nanospheres (Hydrothermal)	Gap	NO_2_	10	20	74%	3.6 />50 min	(52)
MoS_2_ (Commercial Powder) + LIG	Gap	NO_2_	5	150	87%	>20 />33 min	(51)
LIG	Channel	NO	1	60	0.40%	1.8/5.5 min	(54)
LIG + PDMS	Channel	NO	1	60	0.13%	15/47 min	(54)
rGO + MoS_2_	Channel	NO_2_	1	60	0.50%	6/12 min	(53)
SnO (reflux)	Gap	MeOH	150	20	3.70%	50/5 s	This Work

### Sensing Mechanism

The mechanism for VOC detection on SnO at room temperature is challenging to establish experimentally and is therefore not described in detail in the available literature. Chemiresistive MOX sensors are typically considered in terms of an oxygen-mediated process, devised in 1962 by Seiyama et al.^19^ This route typically assumes a high temperature of operation to achieve oxygen radicals or charged oxygen species at more moderate temperatures. This mechanism has received broad adoption due to its prevalence at high temperatures and for noble metal decorated MOX systems, catalyzing oxygen ionization.^25,71,72^ However, it may not be generalizable to room temperature processes. Alternative mechanisms have been proposed, including water-mediated routes and direct adsorption with charge exchange. Water-mediated routes have been proposed at room temperature on Fe_2_O_3_, attributed to the formation of hydroxyl groups mediating the charge exchange.^73^ Similarly, direct adsorption and charge exchange have also been proposed to explain electron withdrawal of NO_*x*_ on MoS_2_ at room temperature.^52^

**Figure 8:**
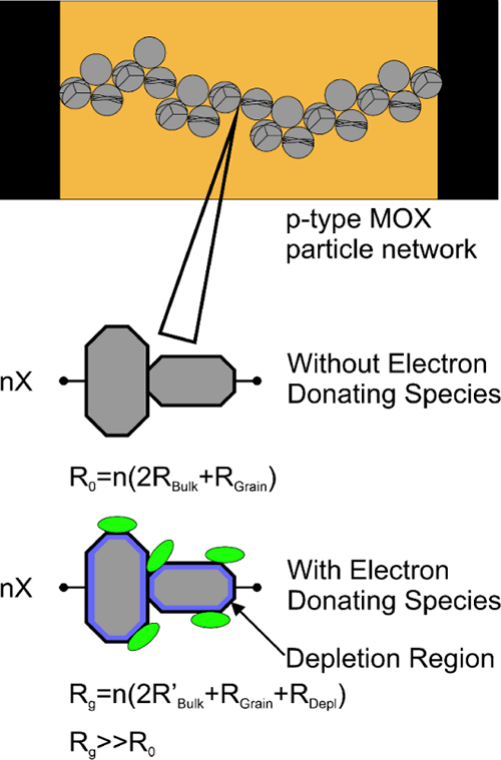
Schematic (not to scale) showing a simplified SnO particle network between two LIG electrodes and the effect of electron donation species on a particle boundary via the generation of a hole depletion layer.

Our data suggest that the chemiresistive SnO response to various reducing VOC gases in this work is unlikely to be dominated by an oxygen-mediated process. First, the humidified nitrogen gas flow used would not provide the oxygen required to replace molecules consumed at the MOX surface in an oxygen-mediated process. Since no significant drift between measurements was observed, the response cannot be explained in terms of adsorbed surface oxygen available at the start of the experiment. Similarly, no significant dependence on the humidity was observed. We therefore propose that the interaction between VOC molecules (MeOH, EtOH, IPA, ACE, and water) arriving at the SnO-MFs is expected to be dominated by adsorption and charge exchange, as depicted in Figure 8.

The surfaces on a typical SnO-MF petal comprise two main crystal orientations: The flat top and bottom surfaces are (001)-oriented and the single- or double-bevel petal edges have (101)-equivalent orientations.^40^ Given the quasiflower structure, SnO-MF assemblies are expected to feature edge–edge and/or edge-surface junctions (Figure 3a) and we expect these edge-junction pinch points to have a significant influence on measured device resistance.

Charge transport through (001)-oriented regions depends on a combination of the petal thickness and the degree of surface charge depletion. In thick petals, we expect bulk *p*-type SnO conduction to dominate, with only a minor contribution from the more resistive surface depletion layer arising from electron donation by adsorbed VOC molecules. In thinner petals, charge transport should be dominated by the depletion layer, either by reducing the effective thickness of the bulk SnO layer or by complete depletion. This depletion layer effect is enhanced in the (101)-oriented petal edges due to their smaller dimensions.

**Figure 9:**
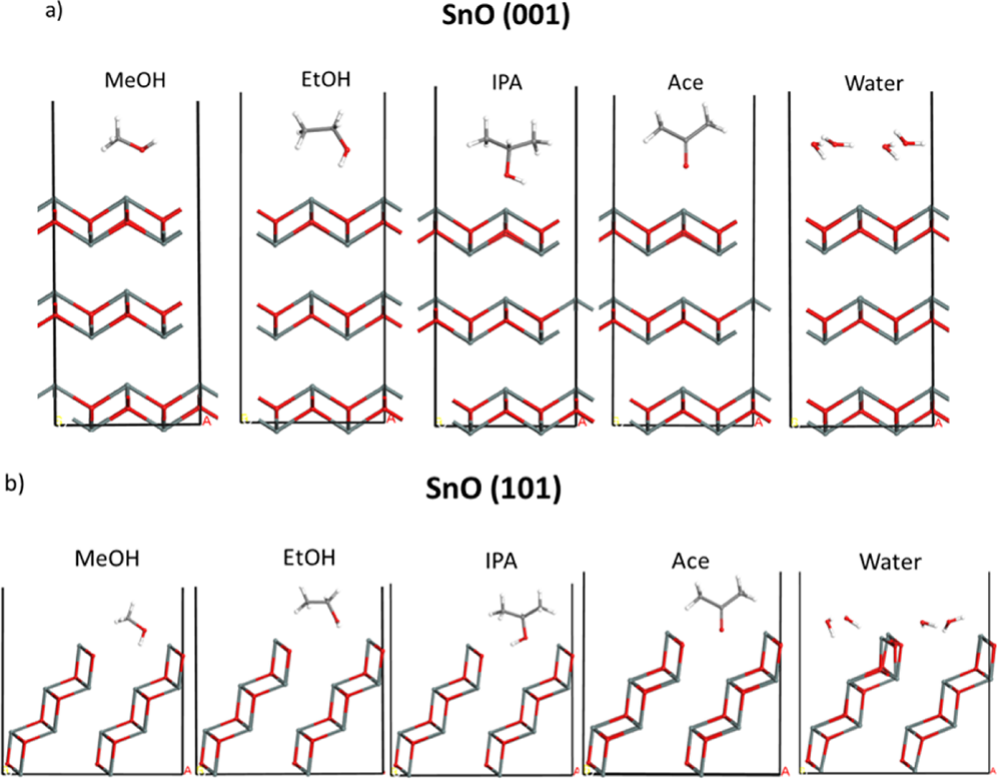
Optimized atomic structures of MeOH, EtOH, IPA, Ace and H_2_O on (a) SnO (001) and (b) SnO (101) surfaces. Color scheme: tin: dark gray, carbon: light gray, oxygen: red, and hydrogen: white.

Density functional theory (DFT) calculations were performed to gain more insight into the interaction of VOC species and associated charge exchange on (001)-oriented SnO (petal surfaces) and also on (101)-oriented SnO (petal edges). Figure 9 shows cross-sectional views of the equilbrium molecular configurations of MeOH, EtOH, IPA, ACE, and water, respectively, at SnO (001) and SnO(101) surfaces. Table 4 shows the computed interaction energy (Δ*E*) values per molecule, with negative values indicating stable interactions for all molecules studied. The magnitudes of the computed interaction energies (0.29 to 0.78 eV) and the absence of a single preferential adsorption site in the equilibrium binding configurations are consistent with physisorption (Figure 9). For the alcohols, the computed interaction energies on SnO (001), 0.29–0.45 eV, are smaller than or comparable to the value for water, 0.43 eV, with MeOH showing the weakest adsorption.

**Table 4:** Computed Molecular Interaction Energy (Δ*E*) for each VOC Molecule and Water on SnO (001) and Sn (101) Surfaces and Net Bader Charge on Surface Sn Atoms (*q*_Sn_) in Units of the Electronic Charge (e^–^) for each Molecule on Both Surfaces

	Surface	Methanol	Ethanol	Isopropanol	Acetone	Water
SnO (001)						
Δ*E* per molecule	–	–0.29 eV	–0.37 eV	–0.45 eV	–0.41 eV	–0.43 eV
Net Sn charge, *q*_Sn_	2.6 e^–^	2.4–2.6 e^–^	2.4–2.6 e^–^	2.5–2.6 e^–^	2.5–2.6 e^–^	2.4–2.6 e^–^
SnO (101)						
Δ*E* per molecule	–	–0.78 eV	–0.64 eV	–0.65 eV	–0.35 eV	–0.50 eV
Net Sn charge, *q*_Sn_	2.3–2.4 e^–^	2.3–2.8 e^–^	2.5–2.9 e^–^	2.5–3.2 e^–^	2.7–3.6 e^–^	2.5–3.0 e^–^

By contrast, all 3 alcohols show significantly larger interaction energies on SnO(101), from 0.65 to 0.78 eV, versus water (0.50 eV), with MeOH showing the strongest adsorption (0.78 eV). The band gap of the SnO (101) surface was estimated from the valence to conduction band energy difference in the density of states (DOS) as ∼1.0 eV. This energy gap persists after the organic molecules and water interact at the surface, further supporting the physisorption scenario.

These data suggest that VOC adsorption on the (101)-oriented edge planes which form edge-junction pinch points between mesoflowers will influence chemiresistive charge transport through-quasiflower devices. The mechanism of ACE is unclear. ACE shows an interaction energy (0.41 eV) that is comparable to that of water on SnO (001), but a slightly lower interaction energy (0.35 eV) on SnO (101).

The edge-junction scenario would also explain the insensitivity of our mesoflower devices to relative humidity since all three alcohols show stronger binding to SnO (101) vs water. It is also worth noting that since the VOC interactions are relatively weak (interaction energies <1 eV), the very high flux of humidified nitrogen used in the purge cycles used should permit surface desorption of these low concentrations of VOC molecules.

Table 4 also shows the net charge on Sn atoms (*q*_Sn_) for both SnO(001) and SnO(101), computed from the Bader partitioning scheme.^74^ For bare SnO(101) *q*_Sn_ = 2.4 e^–^ for surface Sn atoms and *q*_Sn_ = 2.3–2.4 e^–^ for Sn in the first subsurface layer, where e^–^ denotes the electronic charge. Following adsorption of VOC molecules, the net charge for surface Sn atoms increases by ∼0–0.8 e^–^ for the alcohols, ∼0.4–1.2 e^–^ for ACE and ∼0.2–0.8 e^–^ for water. Increased electron donation to the *p*-type SnO would result in an increase in the device resistance, in agreement with measured data. Figure 9 indicates that there appears to be a preferential orientation of the hydroxyl group for each alcohol and the ACE carbonyl group toward the SnO (101) surface, suggesting electron donation from oxygen groups. For the SnO (001) surface, calculations indicate that VOC adsorption does not increase the net charge on the surface Sn atoms.

### Selectivity

The resource-efficient hybrid LIG-SnO VOC sensors presented here demonstrate good sensitivity and stability under ambient conditions. They share the selectivity challenges common to all chemiresistive MOX sensors fabricated by using pristine or undecorated MOX particles. However, reported literature methods to improve selectivity in chemiresistive MOX sensors (including codeposition and decoration with noble metals) are resource-intensive and present sustainability challenges.^26,27,66^ Considering future resource-efficient methods to improve selectivity for our LIG-SnO devices, Jaśkaniec et al. observed that the choice of reflux solvent and temperature determine the morphology of this family of SnO nanoparticles.^40^ For example, butanol reflux led to cubic platelets instead of the mesoflowers produced from the IPA reflux. The DFT results above suggest that using distinct SnO particle morphologies and/or other abundant MOX materials in separate LIG-MOX sensing elements in a multisensor array could improve selectivity by utilizing an interaction matrix integrated with a machine learning layer.^75,76^

### Environmental Considerations

As described earlier (Table 1), we focused on reducing the environmental impact of both the active MOX material in our chemiresistive VOC sensors and also the contact electrodes. For our work, the 3D porous carbon LIG electrodes used are expected to show reduced global warming potential and resource depletion impacts vs lithographically patterned or printed metal contact electrodes. Typical MOX materials used in chemiresistive VOC sensors result in significant environmental burdens during fabrication, including high-temperature processing, see Section SB. Since many of these materials also require high operating temperatures during sensing (>300 °C) operation, this presents additional fabrication burdens, including the need for high-energy ceramics or other thermally stable substrates, as well as integrated heating elements. The solvent–reflux approaches reported by Jaśkaniec et al. and used in this work offer a >3× reduction in synthesis temperature, with associated reductions in environmental footprint.^40^

We also focused on reducing the mass loading of the SnO-MFs, < 25 μg SnO per device, targeting additional reductions in the environmental footprint vs thin or thick MOX films used in conventional chemiresistive VOC sensing devices. As an order of magnitude approximation, taking the density of SnO as 6.45 g/cm^3^ and the device area as ∼2 cm^2^, a mass loading of 25 μg of SnO yields an estimated equivalent film thickness of ∼20 nm.

As shown in Figure 5, there are significant variations in baseline device resistances of nonshorted devices with a measured range ∼300–1800 MΩ, mean resistance μ_R_ ∼ 950 MΩ, standard deviation ∼ 430 MΩ(Figures 5,9), and coefficient of variation = σ_R_/ μ_R_ ∼ 45%. This variation in baseline device resistance is a consequence of the low number of conducting paths present at low loadings of the drop-deposited SnO-MFs. One route to stabilize the baseline resistance is to employ spiral electrode geometries with slightly increased mass loadings (∼50 μg SnO per device); see Section SC.3. Initial investigations using spiral electrode designs with larger gap spacing showed improved yields of functioning, nonshorted devices (12 of 12) vs an approximately a 35% yield (*n* > 200), for narrower-gap two-finger electrodes, see Figure S18. The spiral designs create a ridge-rut geometry along the path of flow for dropcast particles aiding their aggregation and facilitate for multiple conduction paths, e.g., moving from parallel, isolated conduction paths (1 + 1D network) to 2D paths.

The increased gap spacing also resulted in an increased baseline resistance, μ_R_ ∼ 3.1GΩ, however, the coefficient of variation is significantly lower, σ_R_/μ_R_ = 1.0/3.1∼32%. This reduction in variance is likely due to an increase in the number of potential paths due to the spiral geometry, i.e., equivalent to two longer parallel finger electrodes. We note that an increase in the number of potential paths may also reduce the response magnitudes. Narrower gap widths (240 μm, 225 μm) were trialed and showed decreased mean baseline resistance but with increased coefficient of variation and lower yields of functioning, nonshorted devices. Future work will focus on co-optimization of performance and mass loading.

An important consideration for the sustainability of MOX sensors is the useful lifetime of the sensor, t_life_, vs the cumulative energy demand used to fabricate the MOX material, *E*_MOX-Fab_, especially where *E*_MOX-Fab_ represents a substantial contribution to the total cumulative energy demand associated with sensor fabrication. For sensors featuring drop-deposited MOX material of mass m_MOX_, we consider an average fabrication energy per unit mass for a batch-fabrication process, *E*_Batch,m_:
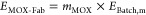
3

We can define the MOX fabrication energy per unit lifetime for each sensor as
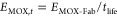
4

**Figure 10:**
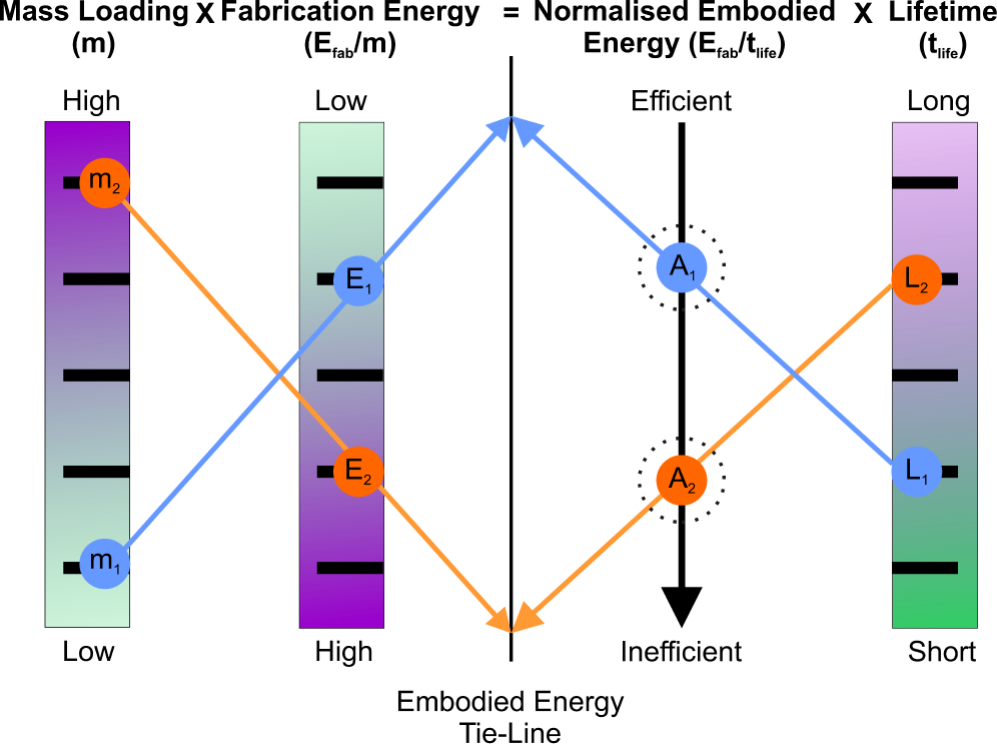
Tie-line schematic for predicting normalized embodied energy given a certain mass loading of an active material to produce a sensor device of estimated working life showing two test cases, described in depth in Section SB.

We can then represent different scenarios using a tie-line approach (Figure 10), which is described in Section SB.1. This suggests that for conventional MOX materials with large fabrication energies and relatively high mass loadings, *E*_MOX,*t*_ will increase, even for longer sensor lifetimes, leading to increased environmental footprint impacts. In addition, MOX sensors that require high-temperature operation will also require increased energy for Joule heating before and during usage cycles, resulting in additional environmental burdens and posing challenges for many applications, e.g., wearable sensors.

Finally, end-of-life scenarios for MOX VOC sensors must also be considered. Efficient recycling pathways are not yet available. Critical raw materials used in electrodes and active materials for conventional MOX sensors (Table 1), e.g., Pt, Pd, Ti, and W, are not recovered, leading to resource depletion. By contrast, our SnO-LIG devices do not contain any critical raw materials. Further, room temperature operation means that these additively fabricated sensors can be formed on glass or plastic support substrates vs alumina or ceramics. We have recently shown that LIG electrodes and sensors can be fabricated using Bioplastic films, which could enable further reductions in environmental footprint impacts for both fabrication and end-of-life life cycle stages.^45,46,50^ Our results highlight the potential for new sensor fabrication and assembly processes to achieve acceptable sensor performance while minimizing environmental footprint, i.e., combined “4-S” co-optimization of sensitivity, selectivity, stability, and sustainability.

## Conclusion

We have developed a resource-efficient route for the fabrication of room-temperature chemiresistive VOC sensors through assembly of sparse SnO-MF networks at laser-graphitized 3D porous carbon (LIG) contact electrodes. Our approach addresses three significant environmental footprint hotspots associated with conventional metal oxide (MOX) VOC sensors: (i) The low-temperature solvent reflux route for these SnO-MFs (<100 °C) offers a >3× reduction in synthesis temperature vs conventional MOX active materials; (ii) direct-written LIG carbon electrodes obviate the need for lithographically patterned or printed metal electrodes; (iii) room temperature operation reduces energy consumption needed for Joule-heating conventional MOX VOC sensors before/during measurements and eliminates the need for, integrated heaters and ceramic substrates.

These SnO-LIG sensors demonstrate responses characteristic of *p*-type semiconductors, with increases in device resistance when exposed to MeOH headspace vapor (∼5% at 150 ppm, ∼35% at 4100 ppm) or other reducing VOCs in a versatile, affordable custom chamber. Density functional theory calculations suggest that the chemiresistive responses are influenced by VOC adsorption on the (101)-oriented edge planes, which form edge-junction pinch points between neighboring SnO-MFs in conduction paths between electrodes.

These SnO-LIG devices yielded a limit of detection of 170 ± 40 ppm for MeOH, below 8-hour workplace safety exposure limits (200 ppm), rapid response and recovery times (*t*_90,resp_ = 50 ± 10 s @150 ppm MeOH, *t*_90,rec_ = 5 ± 0.5 s recovery, *n* = 5) and good stability (>21 days, *n* = 4), competitive with existing resource-intensive chemiresistive MOX sensors.^29,32,77^ Taken together, combinations of LIG electrodes and drop-cast MOX active materials represent a promising resource- and energy-efficient route toward low-power, room-temperature VOC sensors.

## Supporting Information Available

An overview of chemiresistive sensing mechanisms; discussion of SnO chemiresistive sensing mechanism; early stage life cycle analysis motivation; additional experimental setup information, including electrode preparation, gas chamber preparation, gas humidification process, test circuit, and concentration estimation; additional experimental data, including particle size and distribution, UV–vis adsorption spectrum, Mott–Schottky plots, IV characterization, additional XRD characterization, time dependence plots, SEM images indicating device geometry, device failure modes, device reproducibility and alternative electrode geometries, and device aging and stability plots (PDF)
