# Supplementary material for: Room Temperature Sensing of Volatile Organic Compounds Using Hybrid Layered SnO Mesoflowers and Laser-Induced Graphitic Carbon Devices
Source: ACS Sustain Chem Eng. 2024 Sep 27;12(41):15063–76. doi: 10.1021/acssuschemeng.4c04488 (PMC11481091; doi:10.1021/acssuschemeng.4c04488)
Supplement: Supplementary file 1 — sc4c04488_si_001.pdf [file sc4c04488_si_001.pdf]

Supporting Information

Room temperature sensing of volatile organic compounds using hybrid layered SnO mesoflower and Laser-Induced Graphitic carbon devices

Authors

Richard Murray,[a] Arbresha Muriqi,[a] Cathal Larrigy,[a] Alida Russo,[a] Mintesinot Tamiru Mengistu,[a] Daniela Iacopino,[a] Colin Fitzpatrick,[b] Michael Nolan,[a] Aidan J. Quinn[a]*

*Corresponding Author: Aidan.Quinn@Tyndall.ie

[a] Tyndall National Institute, University College Cork, Dyke Parade, Cork T12 R5CP, Ireland

[b] Dept of Electronic & Computer Engineering, University of Limerick, V94 T9PX, Ireland

Number of pages: 34

Number of Figures: 20

Number of Tables: 0

Table of Contents

Supporting Information....1
Authors....1
A – Chemiresistive Sensing Mechanism....3
A.1 Chemiresistive Gas Sensing Theory Overview....3
A.2 SnO for Chemiresistive sensing....4
B – Life Cycle Analysis....8
B.1 Early stage Life Cycle Analysis Motivation....8
C – Gas Sensing Set up....12
C.1 – Sensor assembly and Sampling....12
C.2 3D printed model for sample holder/chamber lid....13
C.3 Humidified Gas Flow....13
C.4 Electrical Sampling Set-up....14
C.5 VOC Concentration Estimates....15
D – Additional Experimental data....16
D.1 Nanoparticle Characterization....16
D.2 Mott-Schottky Measurements....17
D.3 Sensor Time Dependence....20
D.4 Device Structure....21
D.5 Device Stability....23
D.6 Device Aging....28
References....30

A – Chemiresistive Sensing Mechanism

A.1 Chemiresistive Gas Sensing Theory Overview

Semiconducting MOX sensors were initially demonstrated as thin films, although recent advances using nanoparticle morphologies have led to decreased operating temperatures and mass loadings [1,2]. The MOX sensitivity to VOCs derives from the semiconductor conductivity dependence on charge carrier density. When a VOC adsorbs onto the MOX, a charge transfer produces a measurable conductivity change. The mechanism used to describe this is based on early thin film MOX VOC sensors, which described both the direct charge exchange process and a catalytically enhanced oxidation process occurring at elevated temperatures (>400°C) [2,3]. In the oxidation process, adsorbed/ionosorbed oxygen molecules withdraw electrons from the *p*/*n*-type MOX resulting in an increase/decrease of conductivity and producing different anions (O_2^-, O^-, O^{2-}) depending on the temperature. These high-energy charged species may react with VOC molecules and release the withdrawn electrons back into the MOX, undoing their previous modulation, upon exposure to a VOC molecule.

A great variety of MOX materials have been investigated for their sensitivity and selectivity to given VOCs, arising from adsorption site nature and adsorption energies dependences. These include *n*-type semiconducting MOXs, e.g., WO_3 ZnO, SnO_2, TiO_2, In_2O_3 [2–6] and also *p*-type: Co_3O_4, CuO, Cr_2O_3 and NiO [7–10]. To enhance their sensing performance (sensitivity, selectivity, stability, and response/recovery time), several methods have been developed, including decoration with noble metal catalyst particles [11–14]; mixed-deposition of MOX nanoparticles [10,15–19]; morphology changes, including miniaturization [20–22]; high-aspect-ratio structures [23–25]; use of hollow spheres [26–28] and molecularly imprinted polymers [29].

These optimization routes, and combinations thereof, have been widely discussed in the literature [20–22,30–35]. Briefly, metal catalysts and MOX decoration can enhance reactant adsorption and allow spill-

over [36] onto the MOX surface or can open additional reaction routes [37]. Morphological changes affect sensor transduction behavior by modifying charge accumulation or depletion layers and amplifying or attenuating device resistance, respectively, due to increased surface/volume ratios [22]. Similarly, *p-n* junction formation from MOX decoration can also contribute to surface depletion layer formation locally around the junction, producing a conduction route more strongly dependent on gas interaction [17].

However, such optimization routes typically require critical raw materials (e.g., Pt, Rh, Pd, Ti, W) [38], exotic fabrication conditions, and an increase of the complexity of the particle formation process without a significant reduction in device operating temperature. This yields sensor platforms that require significant resources for fabrication and operation, which need to be reduced further if they are to be used extensively as part of the Internet of Things.

Semiconductor MOX nanoparticle investigations have typically favored *n*-type particles, in part due to their greater enhancement in sensing performance with particle size reduction than *p*-type MOX [8]. However, *p*-type materials have recently been demonstrated to have lower optimal operating temperatures than *n*-type MOX; this suggests their suitability for room-temperature VOC sensors [39,40], which would dramatically reduce their energy consumption and improve suitability for wearable sensors. Further, *p*-type responsive sensors are invaluable for machine-learning-enabled e-nose sensor arrays, providing a discrete and opposite response polarity, assisting in the fingerprinting of analytes [41].

A.2 SnO for Chemiresistive sensing

The response of SnO to various reducing gases in this work was conducted using a humidified nitrogen gas flow. The sensing mechanism is unlikely to be oxygen mediated, as the nitrogen gas flow would provide no oxygen to restore those consumed on the MOX surface, and no significant drift between measurements was observed. Similarly, no significant dependence on humidity was

observed. Consequently, the interaction between analyte gases (methanol, ethanol, isopropanol, acetone, and water) and SnO surfaces is expected to be an adsorption/charge exchange-based process.

The petals on the SnO nanoflowers comprise two main crystal orientations: Flat regions (001) planes and the edges (101) planes [42]. These particles aggregate at carbon debris forming a self-assembling SnO nanoflower network between the current collectors, as shown in the SEM micrographs, fig. 4. Due to their layered aggregate nanoflower structure, the granular network of SnO particles deviates from the typical homogenous surfaces of spherical/rod/wire structures implicitly assumed in MOX chemiresistive sensors. Instead, there are numerous particle junctions. Due to the flower morphology, the particles will interface at (101) planes, rather than (001) planes as the petals block the overlap of (001) planes. Therefore the conduction path is comprised of a series of (101) interfaces and parallel paths through (001) top surface and (001) bulk regions. Consequently, the selectivity and degree of interaction of a given analyte will depend on the morphology of the particles used and the relative contributions of these two planes.

The conductivity through the (001) direction depends on a combination of the thickness of the (001) bulk and the degree of surface depletion layer thickness. In large particles, conduction is dominated by the bulk, with only a minor contribution from the more resistive surface depletion layer, caused by electron donation into the SnO. In smaller particles or particles with large surface areas, this conduction is dominated by the depletion layer, either by narrowing the bulk region or by complete depletion. This effect is exaggerated in the (101)-oriented facets of the SnO nanoflowers due to their smaller dimensions in the nanoflowers, resulting in effective depletion layer formation. The network morphology of the sparse SnO nanoflower assemblies suggests that conduction will be mediated through “pinch points” featuring at least one (101)-oriented facet.

To examine the proposed adsorption/charge exchange-based mechanism in more detail, density functional theory (DFT) calculations of the interaction of analyte gases and SnO surfaces were

performed. As SnO is a layered material, surface models of the top nanoflower surface, dominated by the (001) plane, and the nanoflower edges, dominated by the (101) plane, are studied. Interaction energies and the computed net Bader charge on Sn for each analyte-surface combination are presented in Table 3.

Based on the calculated interaction energies all species are expected to interact sufficiently at SnO surfaces at room temperature, with the smallest ΔE being -0.29 eV**.** The range of interaction energies for the analytes are consistent with the sensing ability of SnO, given that they are not so large as to indicate strong adsorption and not so weak that there is no interaction with the surface. No clear single interaction site can be seen in the atomic structures shown in Fig. 8 which is consistent with the magnitude of the interaction energies. There appears to be a preference on both surfaces for the alcohol hydroxyl group and the carbonyl of acetone to be directed to the surface**.**

Therefore the observed humidity independence of the measurements can be explained by our computed interaction energies that indicate no clear preference for water adsorption and water molecules can be displaced by the analyte molecule. The energies and structures can also suggest that water and the analytes will not compete for the same site. Conversely, the computed adsorption energy of water (-0.43 eV/molecule) and its high abundance during the vent can facilitate scouring the surface of other analytes by displacing these species. The larger interaction energy of IPA (-0.45 eV), could partly explain the incomplete removal behavior of IPA shown in Fig. 7a. This can also be caused by IPA being the reaction solvent, which could stabilize the SnO (001) plane and promote preferential IPA binding sites [43].

Different surfaces offer different interaction environments for adsorbing species. The (101) surface shows larger interaction energies than the (001) surface for all species, except acetone. This enhancement is larger for the smaller molecules, e.g. -0.78 vs. -0.29 eV for methanol and -0.65 vs. -0.45 eV for IPA, which is likely associated with the greater steric hindrance of the larger groups, limiting their access to the inter-sheet region. The surface orientation dependence of the interaction energies

means that the sensing response of SnO will be dependent on the contributions of the ensemble of planes involved and this can be useful in motivating further work to control the dominant surface facet of nanostructured SnO.

The band gap of the SnO (101) surface was estimated from the valence to conduction band energy difference in the density of states (DOS) and is *ca.* 1 eV. This energy gap persists after the organic molecules and water interact at the surface, further indicating a non-chemisorption interaction. Typical atomic charges, computed from the Bader partitioning scheme [44], for Sn in the (101) surface layer and the sub-surface layer are 2.4 and 2.3-2.4 electrons respectively. Upon interacting with all molecules, Table 3, the charge of the top layer Sn atoms increases to 2.5-3.6 electrons. This indicates a partial oxidation of surface-terminating Sn species as a result of interactions with the hydroxyl or carbonyl sites. As SnO is a p-type semiconductor, this would increase the resistance due to the associated elimination of hole charge carriers caused by the charge donation when this surface is exposed. By contrast on the (001) surface the change in Sn charges is negligible which is consistent with the weaker interactions between the molecules and this surface. This suggests that the sensing is dominated by the more reactive (101) surface. As already outlined, due to the sparse granular network, the conduction profile is dominated by a series of bottleneck regions of (101) plane interfaces that effectively modulate conduction amplifying the response.

The resource-efficient hybrid LIG-SnO VOC sensors presented here demonstrate good sensitivity and stability under ambient conditions. They share the selectivity challenges common to all chemiresistive MOX sensors fabricated using pristine or undecorated MOX particles. However, reported literature methods to improve selectivity in chemiresistive MOX sensors (including co-deposition and decoration with noble metals) are resource-intensive and present sustainability challenges [45–47]. Considering future resource-efficient methods to improve selectivity for our LIG-SnO devices, *Jaśkaniec et al.* observed that the choice of reflux solvent and temperature determine the morphology of this family of SnO nanoparticles [42]. For example, butanol reflux led to cubic platelets

instead of the nanoflowers produced from IPA reflux. Using distinct SnO particle morphologies to create SnO-LIG sensing elements with known cross-selectivity behavior could enable orthogonal sensing approaches based on multi-sensor arrays on a single substrate. Such sensor arrays, integrated with a machine learning layer, could fingerprint the interaction matrix to enable selective identification, and thus accurate concentration estimation for VOCs [48,49].

B – Life Cycle Analysis

B.1 Early stage Life Cycle Analysis Motivation

Decisions made during the design and development phase determine 80% of all environmental impacts of a given product/process [50–52]. Sustainable design/ecodesign approaches are therefore essential. To fully consider the impacts of a given VOC sensor a full Life Cycle Analysis (LCA) should be conducted. LCA is a class of methods that allows for the comparison of the resource costs and environmental impacts of different means of delivering equivalent functional units. LCA, and related processes, require extensive knowledge about the systems under study, which is particularly difficult during the design of a novel technology due to the immaturity of the technology, small batch fabrication, and difficulty in garnering any concise knowledge about the device's lifetime, use-phase and end of life fate.

A given LCA defines its scope and goal to clarify the parts of the system under study and facilitate cross-comparison and interpretation. The scope refers to the part of the process cycle the study follows, typical studies focus on cradle-to-gate (from when raw materials are extracted until a product is finished), or gate-to-grave (the end-of-life disposal and breakdown of the material) as shown in Figure S1. The goal refers to the measured unit of the process, e.g. 100 measurement hours.

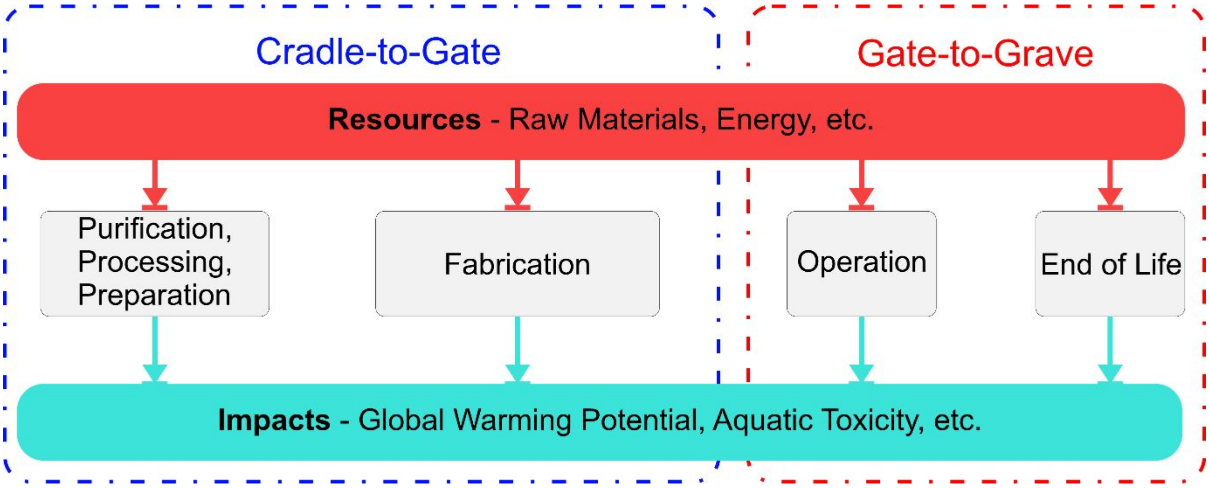

Figure S1. Life Cycle Analysis scope contributing factors.

LCA is a time-consuming process, and the collection of pilot data required for rigorous LCA may not be viable during the decision-rich developmental stages, limiting its application at early stages. Consequently, it is difficult to benchmark a new process against pre-existing devices as much of the current literature lacks LCA consideration, and the materials and methods used advance quickly in the academic literature. This difficulty is amplified by the potential variation in assumptions, system boundaries and functional units which can limit their cross-comparison [51,53].

As a result, most early-stage LCA studies use a streamlined approach and may eliminate life cycle stages, use proxy data, or only consider a limited number of impacts (e.g. Global warming potential or Cumulative Energy Demand), amongst others. Cumulative Energy Demand is considered a particularly useful streamlining approach given the strong correlation between it and other environmental impacts [54]. However, even at a streamlined level LCA remains a specialized skill and requires in-depth knowledge about process routes, materials and origin, equipment, and methods used, which is not always available in sufficient detail in the experimental literature. Consequently, few VOC sensor works provide insight into their environmental impact, despite the increasing societal and environmental importance and impact [53].

Therefore, an initial order of magnitude assessment criteria can provide significant insight into the impact of a VOC sensor, by outlining the broad and dominating contributions at each process step giving an early insight at the design stage. A tie line diagram is included in Fig. 9, to guide this discussion. We present two test cases: 1. A low mass loading of a low energy cost material producing a short lifetime device and 2. A high mass-loading of a high energy-cost active material producing a long lifetime device. The tie-line is used by constructing a line between the mass loading scale point and the Fabrication Energy scale point and projecting it towards the Embodied Energy tie-line. You then connect this intersection with the expected point on the Lifetime scale. The intersection of projected lines and the Normalized Embodied Energy line predicts the energy embodied in a single device accounting for its expected lifetime. This should be compared with the required functional life of the expected use case. The exact details and environmental impacts of each of these considerations in practice would require in-depth consideration as part of an LCA. However, the tie-line approach can be used to pre-screen prospective sensor designs for their environmental impact at the Cradle-to-Gate stage, before and during the sensor design. For instance, the benefit of reducing the mass loading or fabrication energy cost of an active material is self-evident if device lifetime is maintained, showing a reduction in mass normalized Cumulative Energy Demand.

The mass normalized Embodied Energy, as proposed, accounts for the fixed energy costs associated with the fabrication of a device. In addition to the fixed material costs, the high operating temperature required by many chemiresistive VOC sensors is particularly energy intensive and numerous works and approaches have attempted to reduce the temperature required [55]. Therefore, it is necessary to extend this pre-screening approach to the operational life of the sensors. The necessary elevated temperature is typically achieved through integrated Joule heating, an energy intensive process.

The Cumulative Energy Demand (E_{Tot}) can be considered as the sum of both the Cumulative Energy Demand (Fabrication) (E_{Fab}) and the Cumulative Energy Demand (Operation) (E_{Op}). Low operational

temperature is essential for low E_{Op} devices, especially if they have a long lifetime, as the operational energy scales directly with usage. This gives us a proportionality between E_{Tot}, E_{Fab} and E_{Op}, as shown in Equation S1. Similarly, the breakdown of sensors at the end of life can be included as disposal energy (E_{Disp}) to extend the consideration to the Gate-to-Grave.

$$E_{Tot} \propto E_{Fab} + E_{Disp} + E_{Op}(t) \qquad \text{(S1)}$$

The energy demand can therefore be minimized by reduction of the fixed Fabrication Energy or the Operating Energy. Approaches to reduce fabrication energy contributions include: 1) Reduction in the fabrication cost of the active material, 2) Reduction in the extent of active material used, and 3) Elimination of obsolete high energy cost components. Under current sensor approaches, the operational energy, and resulting total energy is dominated by the elevated operating temperature. This has resulted in the development of sensors operating closer to room temperature [55]. As a result, room temperature active materials no longer require thermally robust support materials, such as the typical alumina ceramic supports, or lithographically defined precious metal current collectors and Joule Heaters. Therefore, we eliminate these costly obsolete components in favor of more economical alternatives, reducing the initial resource cost (E_{Fab}), but also disposal cost (E_{Disp}). Therefore, the elimination of high-temperature active material **reduces the operational energy**, by eliminating Joule heating (E_{Oisp} reduced), **reduces the fabrication energy**, by reducing sensor complexity and high energy material dependence (E_{Fab} reduced) and **reduces the disposal energy**, by use of lower impact feedstock and support materials, as sensors are typically not recycled at end of life (E_D reduced) [56].

C – Gas Sensing Set up

C.1 – Sensor assembly and Sampling

As Shown in Figure S2a, sensors were fabricated by dropcast assembly of dilute SnO nanoparticles, synthesized following the methods of Jaśkaniec *et al.* in IPA, on previously prepared LIG electrodes (gap width ≈100 μm) [42]. For each measurement run, up to six dried sensors were mounted in a custom, 3D-printed sample holder (Fig. S3), electrically contacted, and placed into a 600 mL glass flask, see Fig. S2b. Injected solvent was evolved to headspace vapor by heating the chamber base using a hotplate (80°C). Sequential liquid phase additions (Hamilton μL syringe 10 μL, 50 μL, 250 μL) of analyte VOCs and humidified N_2 vents (matched to ambient humidity ~55-65% RH, see S4), were carried out to investigate the VOC sensitivity induced by the SnO nanoflowers. The baseline sensor resistances (~GΩ) exceeded the maximum measurable resistance of the multimeter used (Keithly DAQ 6510, ,100 MΩ detection limit). Therefore parallel balancing resistors (100 MΩ) were used to reduce the overall circuit resistance below the equipment measurement limit (see B.4).

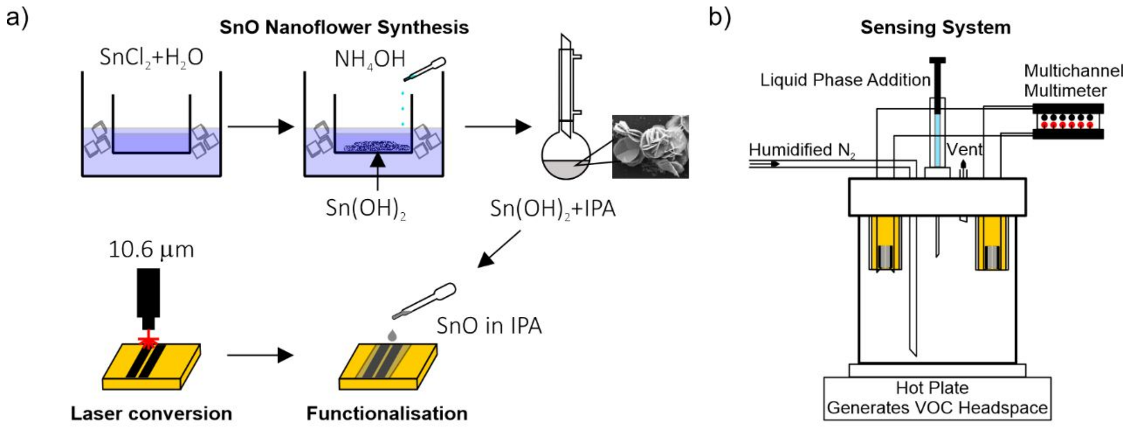

Figure S2. a) Summary process flow for SnO synthesis, Laser-Induced Graphitic carbon formation and drop casting to produce VOC sensors, b) Schematic of VOC sensing apparatus.

C.2 3D printed model for sample holder/chamber lid.

A custom lid for a wide-neck Duran flask (600ml) was modelled and printed from polylactic acid (PLA) on a hobbyist Ender-3 FDM 3D printer. Oversized inset wells for retention of the sample glass slides with adhesive putty, and through holes for coaxial cable, pneumatic tubing and solvent additions were included. This allows for six samples to be monitored simultaneously in the same chemical environment. The associated .stl file is available at: https://github.com/Murray-R/SnO_VOC_Sensor

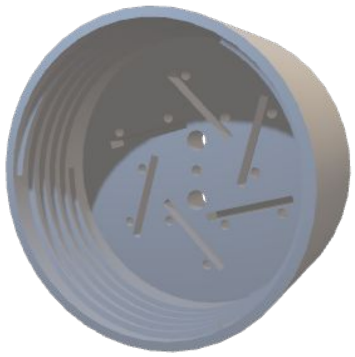

Figure S3. 3D model of printed sample holder-chamber lid.

C.3 Humidified Gas Flow

Fig. S4 depicts the home-built setup used to generate humidified nitrogen for controlled purging of the gas sensing chamber. A bubbler and condenser system were used to humidify dry nitrogen and the gas sensing chamber humidity was adjusted to match laboratory humidity (~45-55% % RH) by

adjusting the flow rate through the dry nitrogen line. Vent cycles lasting 2 minutes with humidified nitrogen flow rates > 20 L/min were used to purge the 0.6 L chamber.

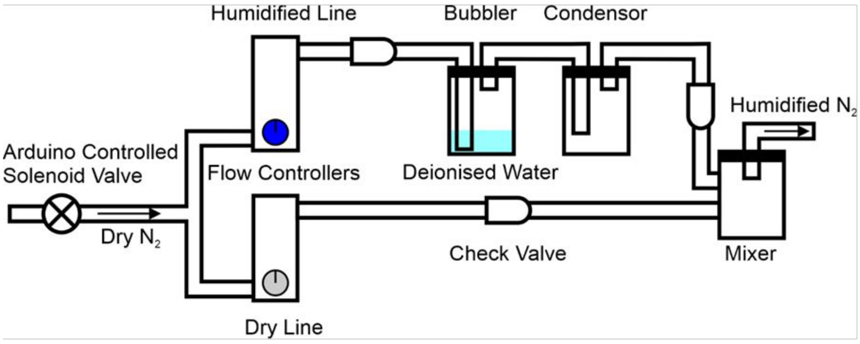

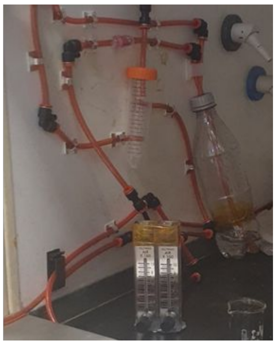

Figure S4. (left) Schematic of humidifying system for nitrogen or air flow. (Right) Image of gas humidifying setup, using a repurposed PET bottle containing deionized water as a bubbler.

C.4 Electrical Sampling Set-up

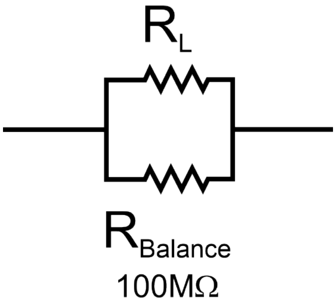

Figure S5. Balancing and load resistor circuit diagram.

$$\frac{1}{R_{Tot}} = \frac{1}{R_{Load}} + \frac{1}{R_{Bal}}$$

$$R_{Load} = \frac{R_{Bal}R_{Tot}}{R_{Bal}-R_{Tot}} \tag{S2}$$

The sensor baseline resistance exceeded the detection limit of the Keithley DAQ6510. A balancing resistor (100 MΩ) was placed parallel to the sensor. The measured total resistance could then be transformed back to determine the sensor resistance using Equation S2.

C.5 VOC Concentration Estimates

The vapor phase concentration of methanol and ethanol were compared to the liquid phase volume additions at different points in time, as shown in fig. S6. The concentration value at 3 minutes after solvent addition was taken as an estimate of the vapor phase concentration which corresponds to the maximum response of the samples. This concentration was verified with a photoionization detector (Tiger 11.7 eV), separately calibrated using calibrated 100 ppm in balance air isobutylene cylinder.

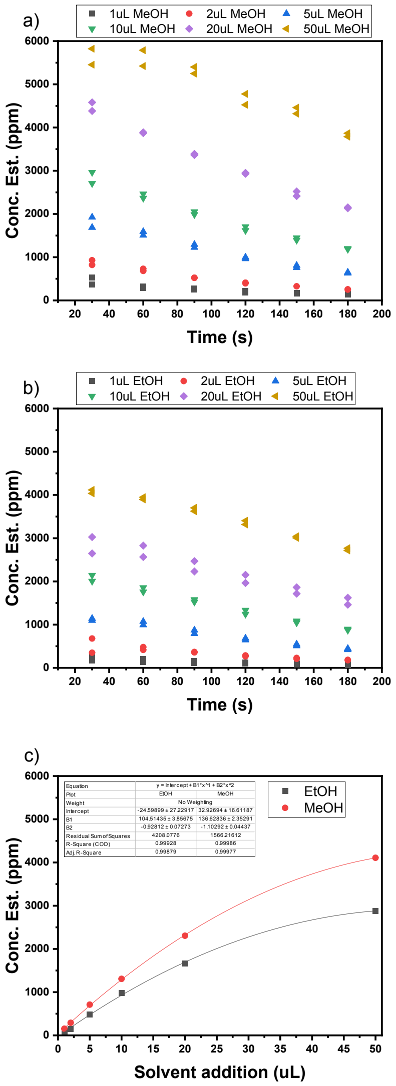

Figure S6. a) Time evolution of different ethanol concentrations with time after addition to sensing chamber, evaporating off a hotplate held at 80°C. b) Time evolution of different methanol concentrations with time after addition to sensing chamber, evaporating off a hotplate held at 80°C. c) Calibration curve of methanol and ethanol concentration (ppm) at 3 minutes after addition against liquid volume addition.

D – Additional Experimental data

D.1 Nanoparticle Characterization

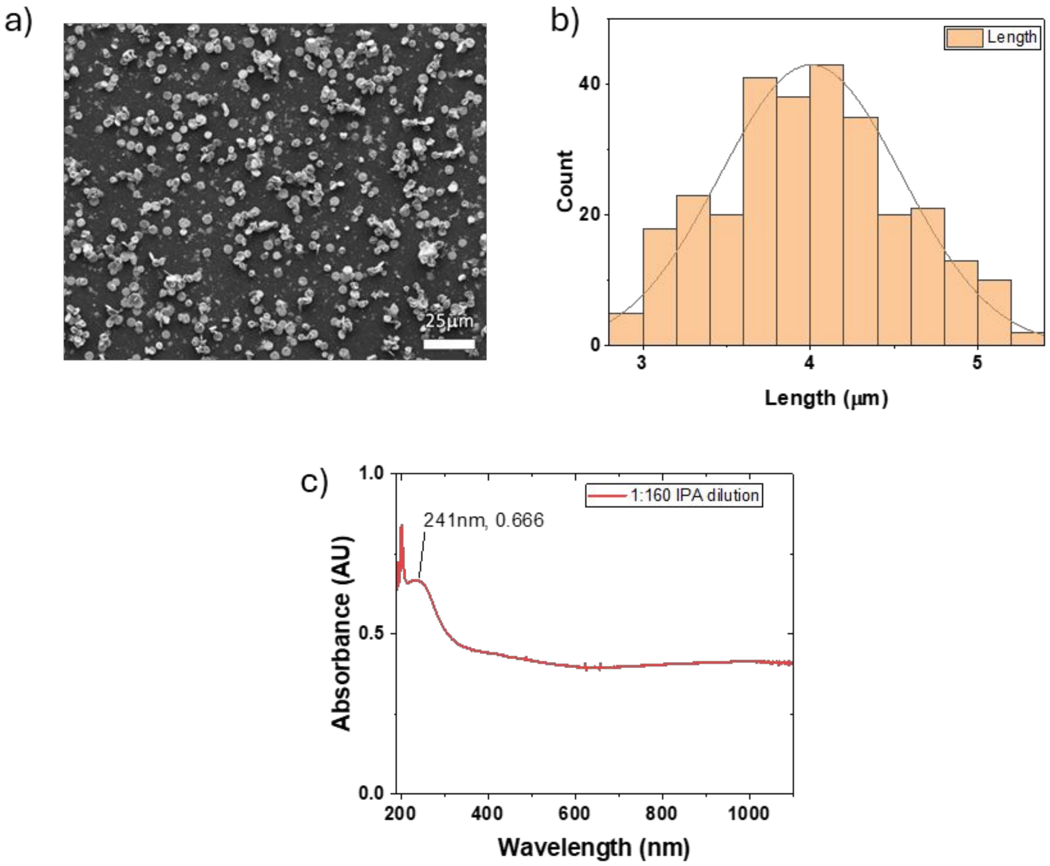

Figure S7. a) Scanning electron micrograph of SnO nanoflowers randomly dispersed on polyimide tape. B) Long-axis particle length distribution showing the diameter of SnO nanoflowers, showing a normal distribution centered around 4µm. c) UV-Vis Adsorption spectrum of SnO particles diluted 1:160 in IPA from as-produced solution, with peak at 241 nm.

Nanoflowers of diameter ≈4 μm were synthesized following the recipe of *Jaśkaniec et al.* with IPA as the reflux solvent, see Fig. S7a,b. Due to the highly concentrated dispersion produced, it was necessary to dilute the dispersion with IPA to measure its absorbance. After a 1:160 dilution, an absorbance of 0.666 at the 241 nm peak was observed. This corresponds to an estimated optical density of 106.6 for the as-produced dispersion and optical density of 13.3, for the 1:8 dilution used for dropcasting onto samples.

D.2 Mott-Schottky Measurements

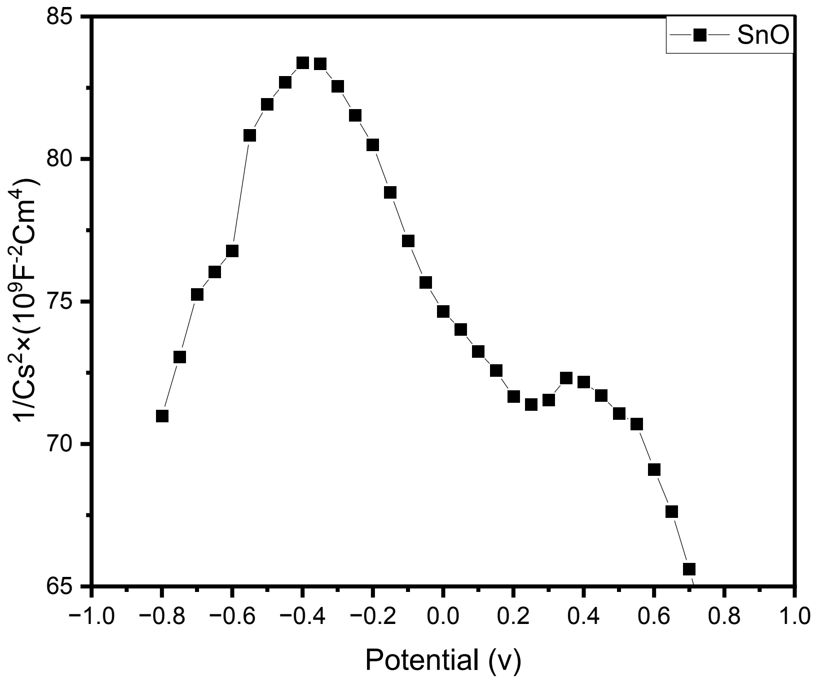

Figure S8. Mott-Schottky plot of SnO nanoparticles on LIG electrode demonstrating p-type semiconductor behavior.

Mott–Schottky measurements were carried out on an electrochemical workstation (CHI760 bi-potentiostat) in 0.5 M KOH Solution. The study was conducted using a three-electrode system, wherein the working electrode was prepared by drop-casting 150 μL of the SnO nanoparticles onto laser-induced graphene. Ag/AgCl and a platinum wire were used as the reference and counter

electrodes, respectively. The potential was scanned from -0.8 to +0.8 V at a frequency of 1 kHz. Figure S8 shows typical data, which demonstrates *p*-type semiconductor behaviour, due to the negative slope with increasing potential [57,58] .

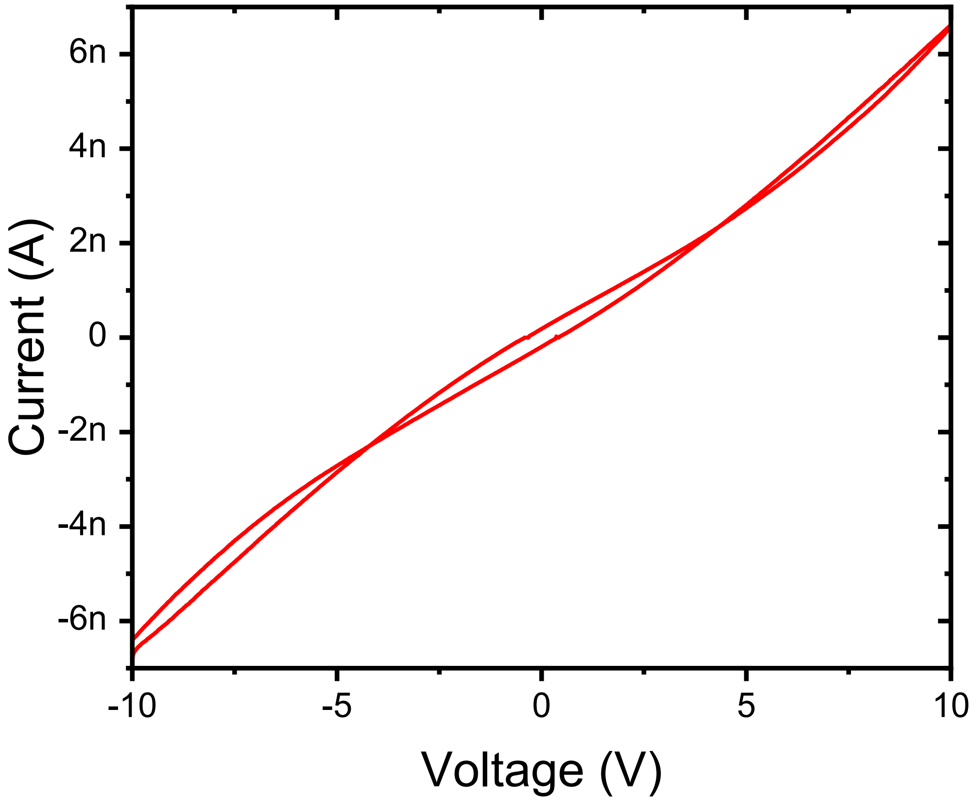

Figure S9. Current Voltage (IV) scan from -10 to 10 V of SnO decorated LIG gap demonstrating non-ohmic contact.

IV scan from -10 to 10 to -10 V was recorded using an Agilent E5270B Parameter Analyzer. This demonstrates non-ohmic behavior, suggestive of a double-Schottky barrier. This was recorded from a spiral patterned LIG electrode, used to reduce the overall system resistance to ensure the system could record the system resistance, given the highly resistive nature of the SnO material.

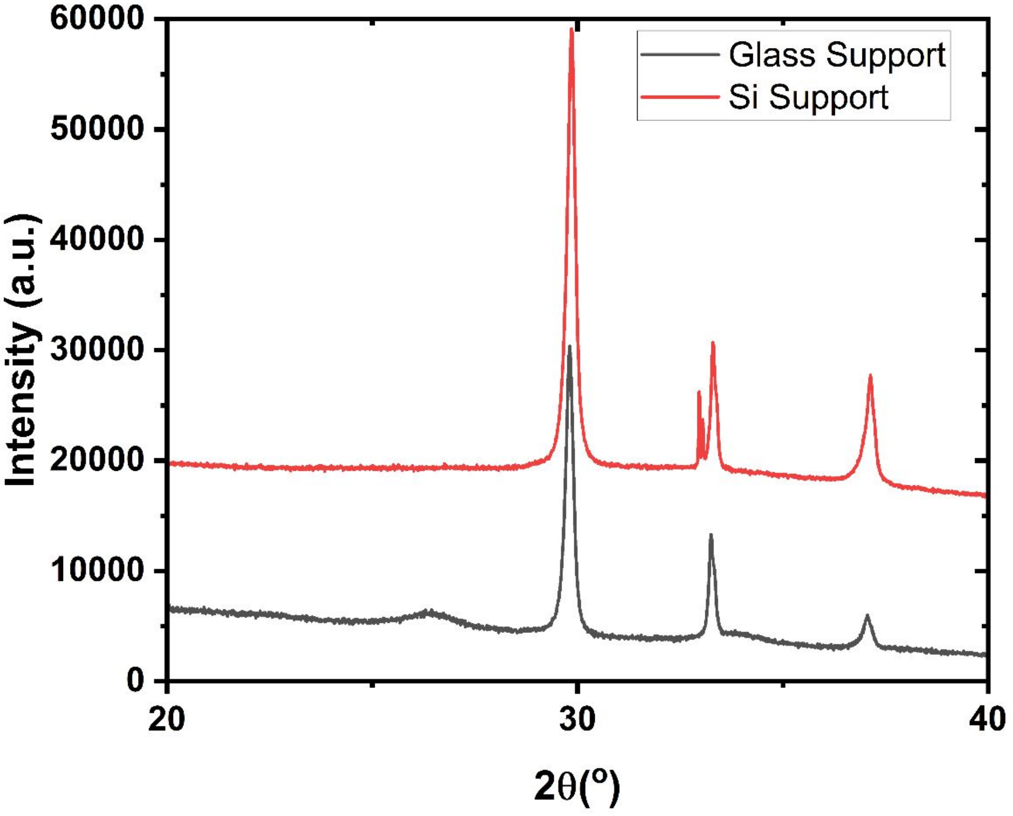

Figure S10. XRD diffractograms of SnO particles on Glass and SI supports.

Figure S10, clarifies the origin of the peak seen at 26.4^{o} as a system peak caused by the glass slide support used to mount the SnO powder for XRD analysis, as its absence can be clearly seen when XRD is repeated for SnO on a Si wafer support. However, typical Si peaks are seen for the Si wafer, which overlap and mask the SnO peaks, preventing its use as a replacement support material.

D.3 Sensor Time Dependence

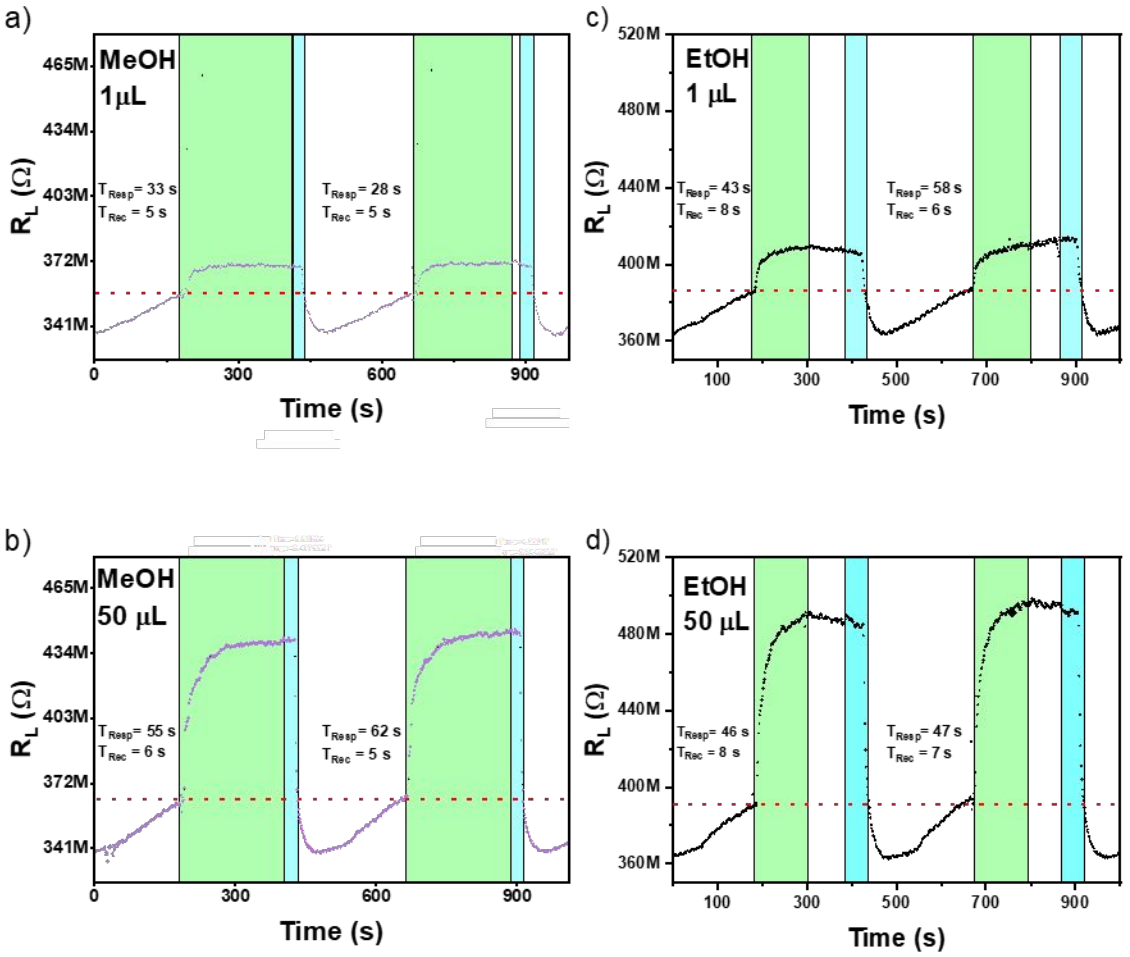

Figure S11. a) Sensor response and recovery time for device D4 (Fig. 5) towards 1 μL (150 ppm at saturation) of MeOH. b) Sensor response and recovery time towards 50 μL (4110 ppm at saturation) of MeOH. c) Sensor response and recovery time for device D4 to 1 μL (90 ppm at saturation) of EtOH. d) Sensor response and recovery time towards 50 μL (2880 ppm at saturation) of EtOH.

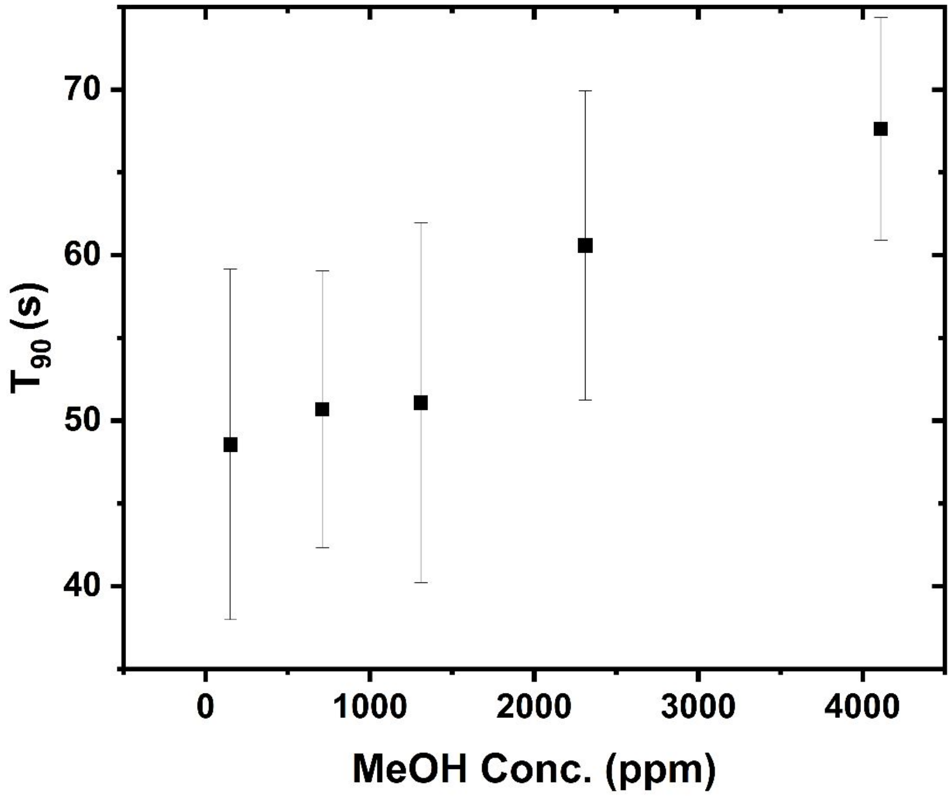

Figure S12. T_{90} Response time of SnO-LIG VOC sensors upon interaction with MeOH at various concentrations, for n=5 devices, and two measurements of each concentration.

D.4 Device Structure

Figure S13 shows different modes of aggregation of SnO nanoflowers and carbon particles between the LIG electrodes. Routes with higher SnO:Carbon ratios, are expected to be more resistive, due to the higher native resistivity of the semiconducting oxide. In practice a sensor would be composed of an assembly of different paths and conformations.

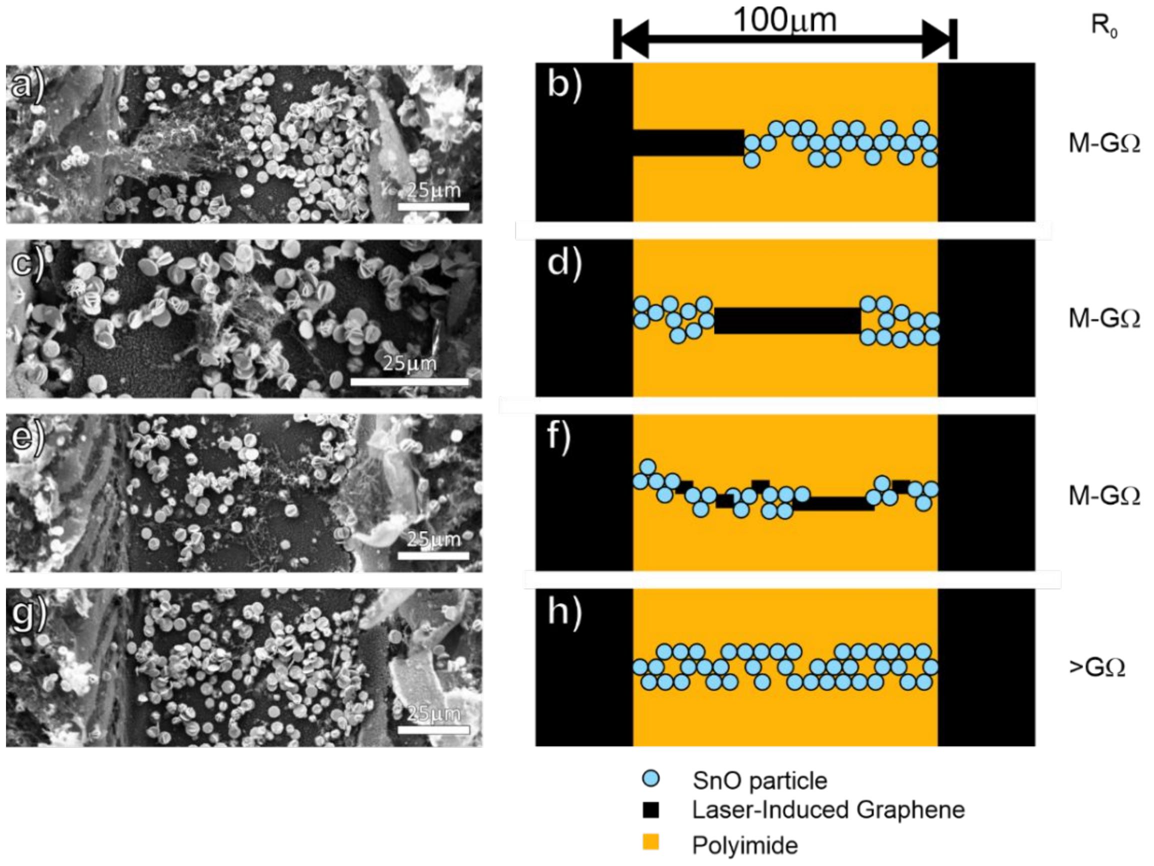

Figure S13. Extended figure for LIG-SnO gap bridging. a) Scanning electron micrograph of partial hybrid carbon bridge-SnO nanoflower mediated conduction channel. b) Schematic of carbon bridge-SnO mediated conduction channel. c) Scanning electron micrograph of central hybrid carbon bridge-SnO nanoflower mediated conduction channel d) Schematic of carbon central bridge-SnO mediated conduction channel. e) Scanning electron micrograph of hybrid carbon islands-SnO nanoflower mediated conduction channel. f) Schematic of carbon islands-SnO mediated conduction channel. g) Scanning electron micrograph of SnO nanoflower mediated conduction channel. h) Schematic of SnO-mediated conduction channel.

D.5 Device Stability

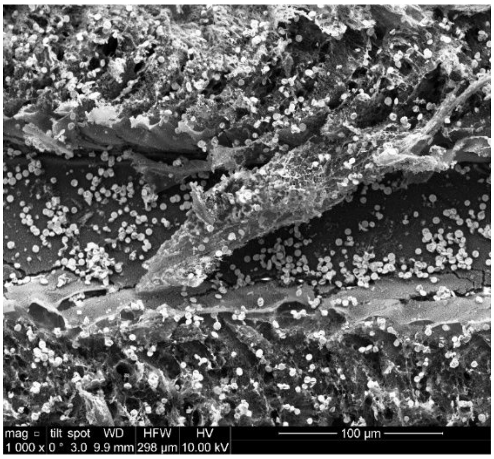

Figure S14. Scanning electron micrograph of full carbon bridging short across the interelectrode gap.

The hybrid LIG-SnO nanoflower devices fall into 3 broad categories: Successful VOC-sensitive devices (R_L ~ MΩ–GΩ), carbon-bridged devices (R_L ~ kΩ) and open circuit devices (R_L >> GΩ). The carbon-bridged devices contain carbon pathways between the electrodes as a by-product of the laser formation process, in addition to the desirable hybrid LIG-SnO pathways. The resistivity of the carbon bridge is not modified by VOC exposure and acts as a low-resistance pathway obscuring any VOC response. A SEM micrograph of this failure mode is shown in Fig. S14. Cleaving of these undesirable conduction paths returns the expected VOC-sensitive resistance of the hybrid-bridge devices, although with increased noise, associated with unintentional damage caused to the SnO pathways.

It is also worth noting that due to the highly resistive nature of SnO an observed "open circuit" can describe two types of devices; a truly open circuit failure mode, and those of resistance above equipment resolution, which dominates this category and is associated with insufficient carbon scaffolding of the SnO aggregation.

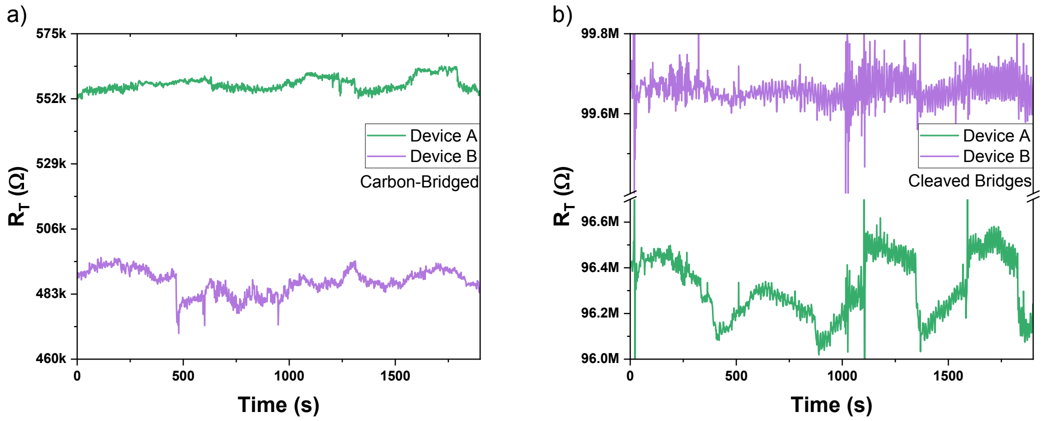

Figure S15. Electrical response behavior towards 2x blank and 2x 5μL methanol VOC exposure of a) fully carbon-bridged VOC sensors and b) the same sensors after the full carbon bridge was cleaved using a scalpel.

The successful VOC-sensitive device resistances varied depending on their combination of bridging structures, ranging from 500 MΩ–1 GΩ. The responses of these successful devices also depend on those bridging structures, as shown in figures S14 & S15. Hybrid conduction networks corresponding to high MΩ–GΩ, figure S16, display a linear response to VOC concentration across the region of interest (0-4000 ppm). The lower resistance devices demonstrate two linear regions, one for low-concentration (<1000 ppm) and one for high-concentration (>1000 ppm). The slope of the low-concentration region of these devices corresponds to those of the higher initial resistance devices, while the high-concentration region demonstrates decreased sensitivity.

Given that the system baseline resistance is defined by the carbon contribution to the bridging network, while the modulation is from the SnO nanoflowers, the sensitivity differences of the two initial resistance regimes are considered. The low-concentration region behaviors of both systems are equivalent, corresponding to highly VOC-sensitive, largely nanoflower-mediated, conduction channels, i.e., 1+1D SnO conduction chains. These structures would exist in both high- and lower-carbon abundance hybrid bridges. The lower initial resistance device mode is due to an increased amount of abundance of carbon-bridging, in the form of higher-density carbon networks or an increased number of carbon islands, facilitating the SnO nanoflower-percolative conduction.

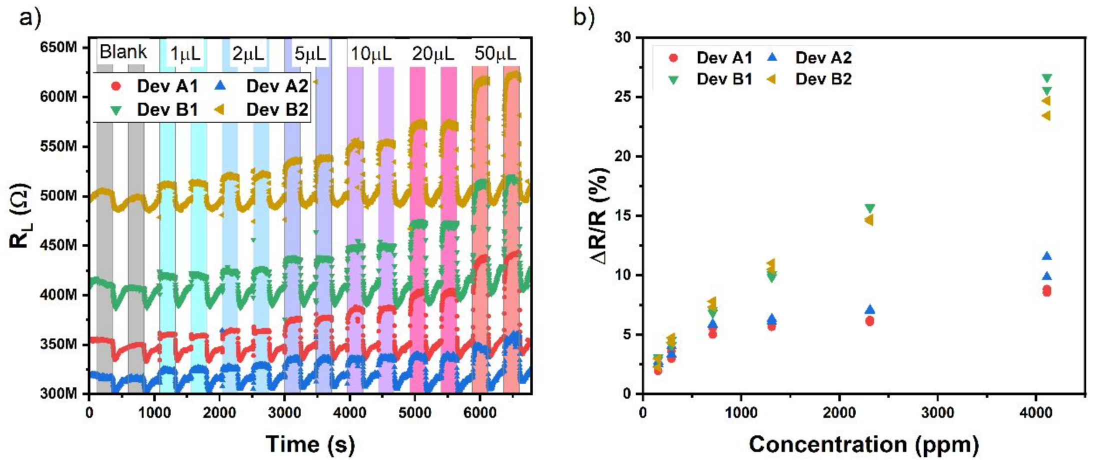

Figure S16. a) Response curve (MeOH) calibration curve of equivalent low initial device resistances (R_L= 300-500 MΩ). b) Extracted calibration curve of equivalent initial resistance devices.

In the lower carbon-concentration hybrid-bridge systems, the 1+1D SnO conduction chains dominate the conduction shutting off parallel pathways of conduction and increasing the resistance monotonically. The higher carbon-concentration system acts as a central conduction node for multiple parallel conduction pathways to proceed from or to cross-link between carbon-bridging structures. This results in a diminished VOC exposure response at higher concentrations as the more critical paths have already been closed.

Unstable sensors have also been observed in this work. These devices still show resistance modulation with exposure to the target VOCs but experience complex behavior during the sampling window. An example of this is shown in Fig. S17, which was recorded at the same time as the samples shown in Fig. 4, but due to the stair-stepping behavior, it could not be treated in the same way without significant processing. These devices demonstrate instability from their first scan, not from an extensive build-up of exposure or cycling, suggesting it is caused by the carbon bridging mechanism.

A variety of carbon-SnO bridging behaviors is possible, the major contributions are shown in Fig. S13. However, the density of the nanoflower density or carbon bridge density and mechanical stability can vary and lead to sensors that deviate from their initial resistance due to carbon pathways

degrading, or densifying of conduction pathways. Due to lack of stable device behavior, such devices are not considered in the characterization of the sensors of this work.

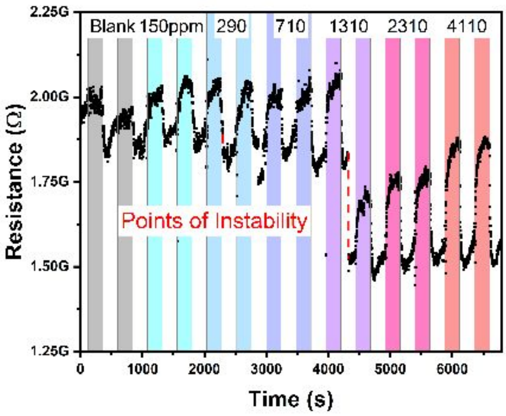

Figure S17. Responsive but unstable gas response behavior of an initially high resistance LIG-SnO hybrid sensor.

This work used a simple two-finger electrode design. This design allows for easier interpretation of the sensing mechanism, however higher device yield and reproducibility would be required for practical adoption. Denser patterns, such as interdigitated designs would provide more parallel paths, reducing the sensor resistance and improving robustness. Due to the dropcast assembly methods used in this work a set of circularly symmetric designs were used. As shown in Fig. S18, these designs have a central dropping region and several LIG tracks at increasing distances to trap the particles in the wells formed. High yields of non-shorted devices were achieved 66-100% were achieved, compared to the ~30% yields demonstrated for simplistic finger designs. Furthermore, the distribution of baseline resistance values were narrowed, with an optimum obtained for the spiral design with a 0.25 mm designed gap between the electrodes. While narrower designed spiral gaps gave some devices with lower baseline resistance and high yield, they possessed a higher variance. This is a fruitful area of optimization for future LIG-based VOC sensors, allowing for highly reproducible sensors, and with

additional optimization tight distributions at controlled baseline resistances.

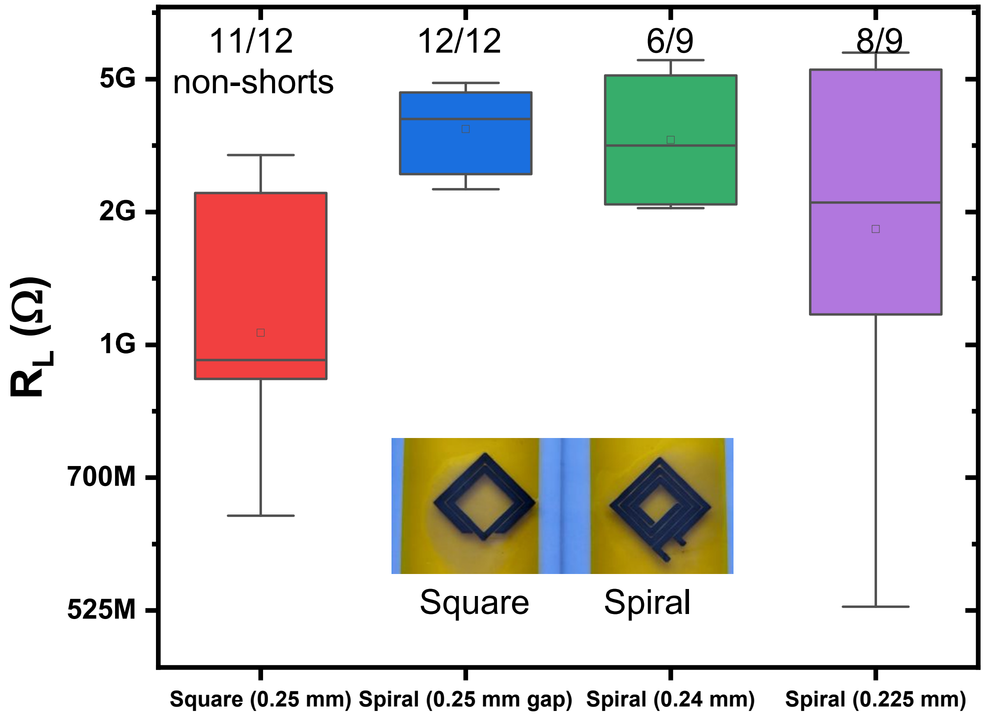

Figure S18. Box plot comparison of the resistances of different electrode morphologies functionalized with SnO Nanoparticles, including functional sensor yield.

D.6 Device Aging

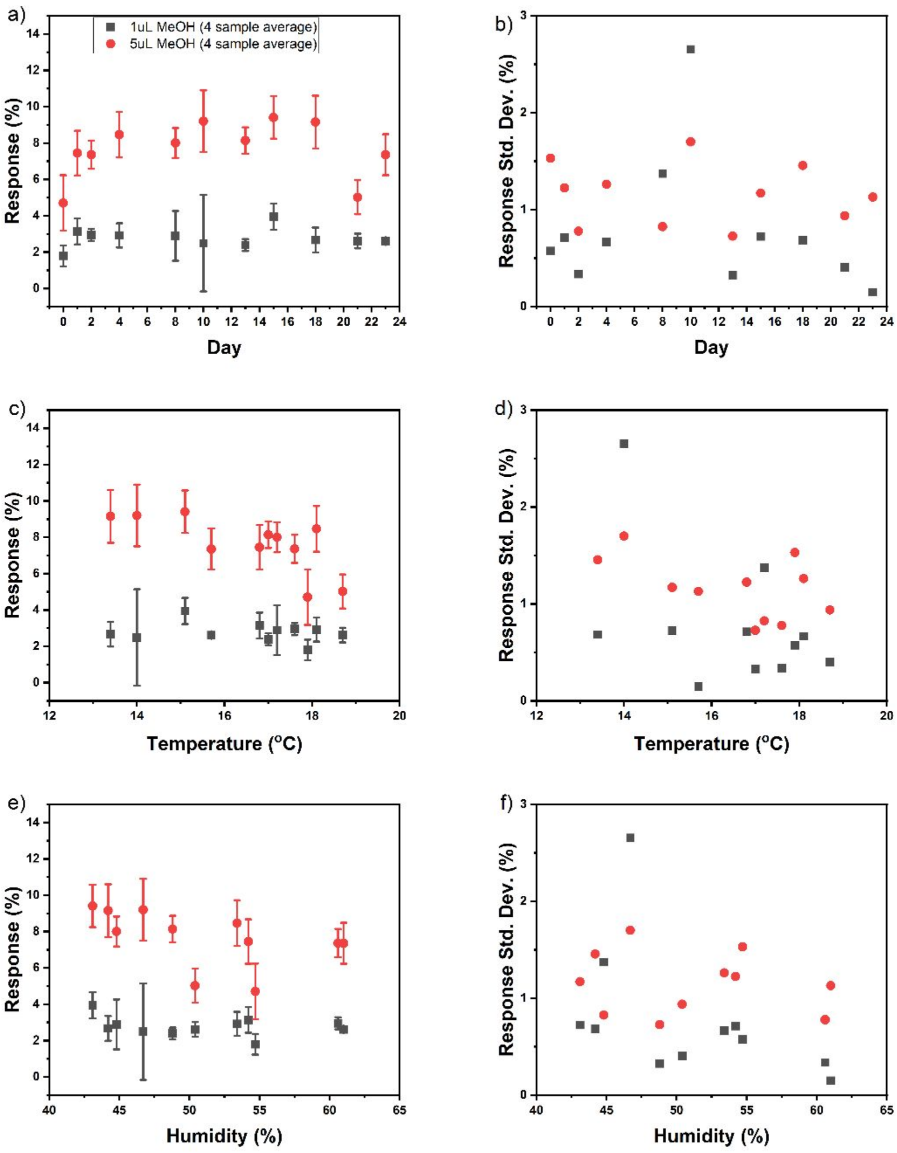

Figure S19. a) Mean (n=4) response stability behavior of SnO-LIG hybrid sensor towards 1 and 5μL of MeOH with aging. b) Mean response variability behavior with gaining. c) Response stability behavior recorded during aging study plotted against ambient room temperature. d) Response variability behavior recorded during aging study plotted against ambient room temperature. e) Means response stability recorded during aging study plotted against room humidity. f) Response variability behavior recorded during aging study plotted against room humidity.

Figure S19 shows an extended version of Fig. 6 demonstrating the aging behavior of 4 devices monitored simultaneously during a series of methanol exposures (2x1 μL and 2x5 μL) on subsequent days. The other two samples in the test chamber didn't behave like typical VOC sensors shown in this work. This is shown in Fig. S20, where device 5 trends downwards in resistance over time and device 6 demonstrates unstable behavior. However, these devices were left in the chamber to ensure the remaining 4 devices would not be affected by opening the system and changing the neighboring devices. The representative, well-behaved, four devices in the chamber were further investigated for the remainder of the aging study.

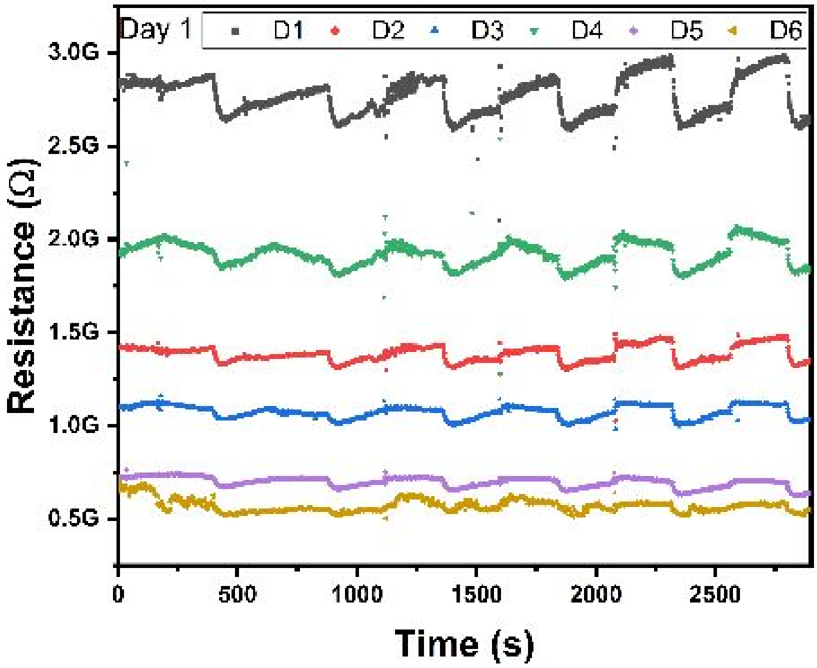

Figure S20. Response behavior of six devices on first measurement run of aging study comprising two blank measurements, two 1μL MeOH additions and two 5μL MeOH additions.

References

(1) Seiyama, T.; Fujiishi, K.; Nagatani, M.; Kato, A. A New Detector for Gaseous Components Using Zinc Oxide Thin Films. *J. Soc. Chem. Ind. Japan* **1963**, *66* (5), 652–655. https://doi.org/10.1246/nikkashi1898.66.5_652.

(2) Shaver, P. J. Activated Tungsten Oxide Gas Detectors. *Appl. Phys. Lett.* **1967**, *11* (8), 255–257. https://doi.org/10.1063/1.1755123.

(3) Seiyama, T.; Kato, A.; Fujiishi, K.; Nagatani, M. A New Detector for Gaseous Components Using Semiconductive Thin Films. *Anal. Chem.* **1962**, *34* (11), 1502–1503. https://doi.org/10.1021/ac60191a001.

(4) Yamazoe, N.; Fuchigami, J.; Kishikawa, M.; Seiyama, T. Interactions of Tin Oxide Surface with O2, H2O AND H2. *Surf. Sci.* **1979**, *86* (C), 335–344. https://doi.org/10.1016/0039-6028(79)90411-4.

(5) Moseley, P. T.; Williams, D. E. A Selective Ammonia Sensor. *Sensors Actuators B. Chem.* **1990**, *1* (1–6), 113–115. https://doi.org/10.1016/0925-4005(90)80183-Z.

(6) Kim, S. J.; Hwang, I. S.; Na, C. W.; Kim, I. D.; Kang, Y. C.; Lee, J. H. Ultrasensitive and Selective C2H5OH Sensors Using Rh-Loaded In2O3 Hollow Spheres. *J. Mater. Chem.* **2011**, *21* (46), 18560–18567. https://doi.org/10.1039/c1jm14252f.

(7) Yoon, J. W.; Choi, J. K.; Lee, J. H. Design of a Highly Sensitive and Selective C2H5OH Sensor Using P-Type Co3O4 Nanofibers. *Sensors Actuators B Chem.* **2012**, *161* (1), 570–577. https://doi.org/10.1016/J.SNB.2011.11.002.

(8) Hübner, M.; Simion, C. E.; Tomescu-Stănoiu, A.; Pokhrel, S.; Bârsan, N.; Weimar, U. Influence of Humidity on CO Sensing with P-Type CuO Thick Film Gas Sensors. *Sensors Actuators, B Chem.* **2011**, *153* (2), 347–353. https://doi.org/10.1016/J.SNB.2010.10.046.

(9) Pokhrel, S.; Simion, C. E.; Quemener, V.; Bârsan, N.; Weimar, U. Investigations of Conduction Mechanism in Cr2O3 Gas Sensing Thick Films by Ac Impedance Spectroscopy and Work Function Changes Measurements. *Sensors Actuators, B Chem.* **2008**, *133* (1), 78–83. https://doi.org/10.1016/J.SNB.2008.01.054.

(10) Wang, C.; Liu, J.; Yang, Q.; Sun, P.; Gao, Y.; Liu, F.; Zheng, J.; Lu, G. Ultrasensitive and Low Detection Limit of Acetone Gas Sensor Based on W-Doped NiO Hierarchical Nanostructure. *Sensors Actuators, B Chem.* **2015**, *220*, 59–67. https://doi.org/10.1016/J.SNB.2015.05.037.

(11) Wang, Z.; Ali Haidry, A.; Xie, L.; Zavabeti, A.; Li, Z.; Yin, W.; Lontio Fomekong, R.; Saruhan, B. Acetone Sensing Applications of Ag Modified TiO2 Porous Nanoparticles Synthesized via Facile Hydrothermal Method. *Appl. Surf. Sci.* **2020**, *533* (July), 147383. https://doi.org/10.1016/j.apsusc.2020.147383.

(12) Yang, X.; Fu, H.; Zhang, L.; An, X.; Xiong, S.; Jiang, X.; Yu, A. Enhanced Gas Sensing Performance Based on the Fabrication of Polycrystalline Ag@TiO2 Core-Shell Nanowires. *Sensors Actuators, B Chem.* **2019**, *286*, 483–492. https://doi.org/10.1016/j.snb.2019.01.096.

(13) Yang, X. H.; Fu, H. T.; Wong, K.; Jiang, X. C.; Yu, A. B. Experimental and Theoretical Studies of V2O5@TiO2 Core-Shell Hybrid Composites with High Gas Sensing Performance towards Ammonia. *Nanotechnology* **2013**, *24* (41), 103–115. https://doi.org/10.1016/j.snb.2017.05.027.

(14) Xing, X.; Chen, N.; Yang, Y.; Zhao, R.; Wang, Z.; Wang, Z.; Zou, T.; Wang, Y. Pt-Functionalized Nanoporous TiO2 Nanoparticles With Enhanced Gas Sensing Performances Toward Acetone. *Phys. Status Solidi Appl. Mater. Sci.* **2018**, *215* (14). https://doi.org/10.1002/PSSA.201800100.

(15) Dutta, P. K.; Ginwalla, A.; Hogg, B.; Patton, B. R.; Chwieroth, B.; Liang, Z.; Gouma, P.; Mills, M.; Akbar, S. Interaction of Carbon Monoxide with Anatase Surfaces at High Temperatures: Optimization of a Carbon Monoxide Sensor. *J. Phys. Chem. B* **1999**, *103* (21), 4412–4422. https://doi.org/10.1021/jp9844718.

(16) Imawan, C.; Solzbacher, F.; Steffes, H.; Obermeier, E. Gas-Sensing Characteristics of Modified-MoO3 Thin Films Using Ti-Overlayers for NH3 Gas Sensors. *Sensors Actuators, B Chem.* **2000**, *64* (1–3), 193–197. https://doi.org/10.1016/S0925-4005(99)00506-7.

(17) Wang, C.; Cheng, X.; Zhou, X.; Sun, P.; Hu, X.; Shimanoe, K.; Lu, G.; Yamazoe, N. Hierarchical α-Fe2O3/NiO Composites with a Hollow Structure for a Gas Sensor. *ACS Appl. Mater. Interfaces* **2014**, *6* (15), 12031–12037. https://doi.org/10.1021/am501063z.

(18) Li, X.; Liu, J.; Guo, H.; Zhou, X.; Wang, C.; Sun, P.; Hu, X.; Lu, G. Au@In2O3 Core–Shell Composites: A Metal–Semiconductor Heterostructure for Gas Sensing Applications. *RSC Adv.* **2014**, *5* (1), 545–551. https://doi.org/10.1039/C4RA12467G.

(19) Kugishima, M.; Shimanoe, K.; Yamazoe, N. C2H4O Sensing Properties for Thick Film Sensor Using La2O3-Modified SnO2. *Sensors Actuators, B Chem.* **2006**, *118* (1–2), 171–176. https://doi.org/10.1016/J.SNB.2006.04.053.

(20) Barsan, N.; Simion, C.; Heine, T.; Pokhrel, S.; Weimar, U. Modeling of Sensing and Transduction for P-Type Semiconducting Metal Oxide Based Gas Sensors. *J. Electroceramics* **2010**, *25* (1), 11–19. https://doi.org/10.1007/s10832-009-9583-x.

(21) Yoon, J. W.; Kim, H. J.; Kim, I. D.; Lee, J. H. Electronic Sensitization of the Response to C2H5OH of P-Type NiO Nanofibers by Fe Doping. *Nanotechnology* **2013**, *24* (44). https://doi.org/10.1088/0957-4484/24/44/444005.

(22) Barsan, N.; Weimar, U. Conduction Model of Metal Oxide Gas Sensors. *J. Electroceramics* **2001**, *7* (3), 143–167. https://doi.org/10.1023/A:1014405811371.

(23) Zheng Wei Pan; Zu Rong Dai; Zhong Lin Wang. Nanobelts of Semiconducting Oxides. *Science (80-.).* **2001**, *291* (5510), 1947–1949. https://doi.org/10.1126/SCIENCE.1058120.

(24) Law, M.; Kind, H.; Messer, B.; Kim, F.; Yang, P. Photochemical Sensing of NO 2 with SnO 2 Nanoribbon Nanosensors at Room Temperature**. *Angew. Chem. Int. Ed* **2002**, *41* (13). https://doi.org/10.1002/1521-3773(20020703)41:13<2405::AID-ANIE2405>3.0.CO;2-3.

(25) Comini, E.; Faglia, G.; Sberveglieri, G. Stable and Highly Sensitive Gas Sensors Based on Semiconducting Oxide Nanobelts. *Appl. Phys. Lett* **2002**, *81*, 1869. https://doi.org/10.1063/1.1504867.

(26) Kim, S. J.; Hwang, I. S.; Choi, J. K.; Kang, Y. C.; Lee, J. H. Enhanced C2H5OH Sensing Characteristics of Nano-Porous In2O3 Hollow Spheres Prepared by Sucrose-Mediated Hydrothermal Reaction. *Sensors Actuators B Chem.* **2011**, *155* (2), 512–518. https://doi.org/10.1016/J.SNB.2010.12.055.

(27) Gyger, F.; H€, M.; Feldmann, C.; Barsan, N.; Weimar, U. Nanoscale SnO 2 Hollow Spheres and Their Application as a Gas-Sensing Material. *Chem. Mater* **2010**, *22*, 4821. https://doi.org/10.1021/cm1011235.

(28) Zhong, Z.; Yin, Y.; Gates, B.; Xia, Y. Preparation of Mesoscale Hollow Spheres of TiO2 and SnO2

by Templating against Crystalline Arrays of Polystyrene Beads. *Adv. Mater.* **2000**, *12* (3), 206–209. https://doi.org/10.1002/(SICI)1521-4095(200002)12:3<206::AID-ADMA206>3.0.CO;2-5.

(29) Song, W.; Zhang, M.; Zhao, W.; Zhao, Q.; Hao, H.; Lin, H.; Gao, W.; Yan, S. Nanostructured SnO2Microsphere-Based Gas Sensor Array Enhanced by Molecular Imprinting for Methanol and Ethanol Discriminative Detection. *ACS Appl. Nano Mater.* **2022**, *5* (9), 12765–12777. https://doi.org/10.1021/acsanm.2c02662.

(30) Lee, J. H. Gas Sensors Using Hierarchical and Hollow Oxide Nanostructures: Overview. *Sensors Actuators B Chem.* **2009**, *140* (1), 319–336. https://doi.org/10.1016/J.SNB.2009.04.026.

(31) Kolmakov, A.; Moskovits, M. CHEMICAL SENSING AND CATALYSIS BY ONE-DIMENSIONAL METAL-OXIDE NANOSTRUCTURES. *Annu. Rev. Mater. Res* **2004**, *34*, 151–180. https://doi.org/10.1146/annurev.matsci.34.040203.112141.

(32) Moseley, P. T. Solid State Gas Sensors. *Meas. Sci. Technol.* **1997**, *8* (3), 223–237. https://doi.org/10.1088/0957-0233/8/3/003.

(33) Yamazoe, N.; Shimanoe, K. New Perspectives of Gas Sensor Technology. *Sensors Actuators, B Chem.* **2009**, *138* (1), 100–107. https://doi.org/10.1016/J.SNB.2009.01.023.

(34) Joshi, N.; Hayasaka, T.; Liu, Y.; Liu, H.; Oliveira, O. N.; Lin, L. A Review on Chemiresistive Room Temperature Gas Sensors Based on Metal Oxide Nanostructures, Graphene and 2D Transition Metal Dichalcogenides. *Microchim. Acta* **2018**, *185* (4). https://doi.org/10.1007/s00604-018-2750-5.

(35) Zhang, J.; Liu, X.; Neri, G.; Pinna, N. Nanostructured Materials for Room-Temperature Gas Sensors. *Adv. Mater.* **2016**, *28* (5), 795–831. https://doi.org/10.1002/adma.201503825.

(36) Bond, G. C.; Molloy, L. R.; Fuller, M. J. Oxidation of Carbon Monoxide over Palladium-Tin(IV) Oxide Catalysts: An Example of Spillover Catalysis. *J. Chem. Soc. Chem. Commun.* **1975**, No. 19, 796–797. https://doi.org/10.1039/C39750000796.

(37) Großmann, K.; Wicker, S.; Weimar, U.; Barsan, N. Impact of Pt Additivies on the Surface Reactions between SnO2, Water Vapour, CO and H2; an Operando Investigation. *Phys. Chem. Chem. Phys* **2013**, *15*, 19151–19158. https://doi.org/10.1039/c3cp52782d.

(38) Blengini, G. A.; Latunussa, C. E. L.; Eynard, U.; Torres de Matos, C.; Wittmer, D.; Georgitzikis, K.; Pavel, C.; Carrara, S.; Mancini, L.; Unguru, M.; Blagoeva, D.; Mathieux, F.; Pennington, D. *Study on the EU's List of Critical Raw Materials (2020) Final Report*; 2020. https://doi.org/10.2873/904613.

(39) Wisitsoraat, A.; Tuantranont, A.; Comini, E.; Sberveglieri, G.; Wlodarski, W. Characterization of N-Type and p-Type Semiconductor Gas Sensors Based on NiOx Doped TiO2 Thin Films. *Thin Solid Films* **2009**, *517* (8), 2775–2780. https://doi.org/10.1016/j.tsf.2008.10.090.

(40) Kwon, H. I. Low-Temperature-Operated Sensitive NO2gas Sensors Based on p-Type SnO Thin-Film and Thin-Film Transistors. *Proc. AM-FPD 2020 - 27th Int. Work. Act. Flatpanel Displays Devices TFT Technol. FPD Mater.* **2020**, No. 2, 55–58. https://doi.org/10.23919/AM-FPD49417.2020.9224482.

(41) Wang, J.; Yang, P.; Wei, X. High-Performance, Room-Temperature, and No-Humidity-Impact Ammonia Sensor Based on Heterogeneous Nickel Oxide and Zinc Oxide Nanocrystals. *ACS Appl. Mater. Interfaces* **2015**, *7* (6), 3816–3824. https://doi.org/10.1021/am508807a.

(42) Jaśkaniec, S.; Kavanagh, S. R.; Coelho, J.; Ryan, S.; Hobbs, C.; Walsh, A.; Scanlon, D. O.; Nicolosi, V. Solvent Engineered Synthesis of Layered SnO for High-Performance Anodes. *npj 2D Mater.*

Appl. 2021 51 **2021**, *5* (1), 1–9. https://doi.org/10.1038/s41699-021-00208-1.

(43) Ren, Q.; Zhang, X.; Wang, Y.; Xu, M.; Wang, J.; Tian, Q.; Jia, K.; Liu, X.; Sui, Y.; Liu, C.; Yun, J.; Yan, J.; Zhao, W.; Zhang, Z. Shape-Controlled and Stable Hollow Frame Structures of SnO and Their Highly Sensitive NO2 Gas Sensing. *Sensors Actuators B Chem.* **2021**, *340*, 129940. https://doi.org/10.1016/J.SNB.2021.129940.

(44) Henkelman, G.; Arnaldsson, A.; Jónsson, H. A Fast and Robust Algorithm for Bader Decomposition of Charge Density. *Comput. Mater. Sci.* **2006**, *36* (3), 354–360. https://doi.org/10.1016/J.COMMATSCI.2005.04.010.

(45) Saji, K. J.; Venkata Subbaiah, Y. P.; Tian, K.; Tiwari, A. P-Type SnO Thin Films and SnO/ZnO Heterostructures for All-Oxide Electronic and Optoelectronic Device Applications. *Thin Solid Films* **2016**, *605*, 193–201. https://doi.org/10.1016/J.TSF.2015.09.026.

(46) Degler, D.; Rank, S.; Müller, S.; Pereira De Carvalho, H. W.; Grunwaldt, J. D.; Weimar, U.; Barsan, N. Gold-Loaded Tin Dioxide Gas Sensing Materials: Mechanistic Insights and the Role of Gold Dispersion. *ACS Sensors* **2016**, *1* (11), 1322–1329. https://doi.org/10.1021/acssensors.6b00477.

(47) Hübner, M.; Bârsan, N.; Weimar, U. Influences of Al, Pd and Pt Additives on the Conduction Mechanism as Well as the Surface and Bulk Properties of SnO2 Based Polycrystalline Thick Film Gas Sensors. *Sensors Actuators B Chem.* **2012**, *171–172*, 172–180. https://doi.org/10.1016/J.SNB.2012.02.080.

(48) Thai, N. X.; Tonezzer, M.; Masera, L.; Nguyen, H.; Duy, N. Van; Hoa, N. D. Multi Gas Sensors Using One Nanomaterial, Temperature Gradient, and Machine Learning Algorithms for Discrimination of Gases and Their Concentration. *Anal. Chim. Acta* **2020**, *1124*, 85–93. https://doi.org/10.1016/j.aca.2020.05.015.

(49) Itoh, T.; Koyama, Y.; Shin, W.; Akamatsu, T.; Tsuruta, A.; Masuda, Y.; Uchiyama, K. Selective Detection of Target Volatile Organic Compounds in Contaminated Air Using Sensor Array with Machine Learning: Aging Notes and Mold Smells in Simulated Automobile Interior Contaminant Gases. *Sensors (Switzerland)* **2020**, *20* (9). https://doi.org/10.3390/s20092687.

(50) Kulatunga, A. K.; Karunatilake, N.; Weerasinghe, N.; Ihalawatta, R. K. Sustainable Manufacturing Based Decision Support Model for Product Design and Development Process. *Procedia CIRP* **2015**, *26*, 87–92. https://doi.org/10.1016/j.procir.2015.03.004.

(51) Keoleian, G. A.; Menerey, D. Life Cycle Design Guidance Manual: Environmental Requirements and the Product System. **1993**. https://doi.org/10.7302/22093.

(52) Chebaeva, N.; Lettner, M.; Wenger, J.; Schöggl, J. P.; Hesser, F.; Holzer, D.; Stern, T. Dealing with the Eco-Design Paradox in Research and Development Projects: The Concept of Sustainability Assessment Levels. *J. Clean. Prod.* **2021**, *281*. https://doi.org/10.1016/j.jclepro.2020.125232.

(53) Hetherington, A. C.; Borrion, A. L.; Griffiths, O. G.; McManus, M. C. Use of LCA as a Development Tool within Early Research: Challenges and Issues across Different Sectors. *Int. J. Life Cycle Assess.* **2014**, *19* (1), 130–143. https://doi.org/10.1007/s11367-013-0627-8.

(54) Huijbregts, M. A. J.; Hellweg, S.; Frischknecht, R.; Hendriks, H. W. M.; Hungehbühler, K.; Hendriks, A. J. Cumulative Energy Demand as Predictor for the Environmental Burden of Commodity Production. *Environ. Sci. Technol.* **2010**, *44* (6), 2189–2196. https://doi.org/10.1021/es902870s.

(55) Majhi, S. M.; Mirzaei, A.; Kim, H. W.; Kim, S. S.; Kim, T. W. Recent Advances in Energy-Saving

Chemiresistive Gas Sensors: A Review. *Nano Energy* **2021**, *79* (September 2020), 105369. https://doi.org/10.1016/j.nanoen.2020.105369.

(56) Modarress Fathi, B.; Ansari, A.; Ansari, A. Threats of Internet-of-Thing on Environmental Sustainability by E-Waste. *Sustain.* **2022**, *14* (16). https://doi.org/10.3390/su141610161.

(57) Bera, B.; Chakraborty, A.; Kar, T.; Leuaa, P.; Neergat, M. Density of States, Carrier Concentration, and Flat Band Potential Derived from Electrochemical Impedance Measurements of N-Doped Carbon and Their Influence on Electrocatalysis of Oxygen Reduction Reaction. *J. Phys. Chem. C* **2017**, *121* (38), 20850–20856. https://doi.org/10.1021/acs.jpcc.7b06735.

(58) Brinker, M.; Huber, P. A Mott-Schottky Analysis of Mesoporous Silicon in Aqueous Electrolyte Solution by Electrochemical Impedance Spectroscopy. *Electrochim. Acta* **2024**, *483*, 1–7. https://doi.org/10.1016/j.electacta.2024.144038.
